# DNA methylation signatures of bilateral hippocampal volume, asymmetry and atrophy: a cross-omics analysis in the general population

**DOI:** 10.1016/j.ebiom.2026.106289

**Published:** 2026-05-11

**Authors:** Dan Liu, Valentina Talevi, Juliana F. Tavares, Ruiqi Wang, Mohammed A. Imtiaz, Konstantinos Melas, Alexander Teumer, Katharina Wittfeld, Robert F. Hillary, Dina Vojinovic, Marian Beekman, Nicola J. Armstrong, Santiago Estrada, Henry Völzke, Robin Bülow, Natalie A. Royle, Joanna M. Wardlaw, Wei Wen, Perminder S. Sachdev, Karen A. Mather, P. Eline Slagboom, Simon R. Cox, Hans Jörgen Grabe, Qiong Yang, N. Ahmad Aziz, Monique M.B. Breteler

**Affiliations:** a Population Health Sciences, German Centre for Neurodegenerative Diseases (DZNE), 53127, Bonn, Germany; b Department of Biostatistics, Boston University School of Public Health, Boston, MA, 02118, USA; c Department of Psychiatry and Psychotherapy, University Medicine Greifswald, 17489, Greifswald, Germany; d German Centre for Cardiovascular Research (DZHK), Partner Site Greifswald, Greifswald, 17475, Germany; e Lothian Birth Cohorts, Department of Psychology, The University of Edinburgh, Edinburgh, EH8 9JZ, UK; f Molecular Epidemiology, Department of Biomedical Data Science, Leiden University Medical Centre, 233 ZA, Leiden, the Netherlands; g Mathematics and Statistics, Curtin University, 6845, Perth, Australia; h Artificial Intelligence in Medical Imaging, German Centre for Neurodegenerative Diseases (DZNE), 53127, Bonn, Germany; i Institute for Community Medicine, University Medicine Greifswald, 17489, Greifswald, Germany; j Institute of Diagnostic Radiology and Neuroradiology, University Medicine Greifswald, 17489, Greifswald, Germany; k Brain Research Imaging Centre, The University of Edinburgh, Edinburgh, EH8 9AB, UK; l Centre for Healthy Brain Ageing, Discipline of Psychiatry and Mental Health, School of Clinical Medicine, University of New South Wales, Sydney, NSW, 2052, Australia; m Max Planck Institute for Biology of Ageing, 50931, Cologne, Germany; n German Centre for Neurodegenerative Diseases (DZNE), Partner Site Rostock/Greifswald, 17489, Greifswald, Germany; o The Framingham Heart Study, Framingham, MA, 01701, USA; p Department of Neurology, Faculty of Medicine, University of Bonn, 53127, Bonn, Germany; q Institute for Medical Biometry, Informatics and Epidemiology (IMBIE), Faculty of Medicine, University of Bonn, 53127, Bonn, Germany

**Keywords:** Biomarker, DNA methylation, Hippocampal volume, Hippocampal asymmetry, Diet, Population-based

## Abstract

**Background:**

Left-right hippocampal volumetric asymmetry and atrophy are implicated in neurodegenerative and neuropsychiatric disorders, yet their molecular basis in healthy adults remains poorly understood.

**Methods:**

We conducted a meta-analysis of epigenome-wide association studies across six population-based cohorts (n = 8156; 53% women; mean age = 60.7 years) to identify DNA methylation signatures associated with left and right hippocampal volumes (LHCV, RHCV) and hippocampal asymmetry (i.e, differences between left and right volumes divided by their sums).

**Findings:**

We identified five CpGs and 262 differentially methylated regions associated with LHCV, nine CpGs and 246 regions with RHCV, one CpG and 16 regions with asymmetry. Cross-omics integration uncovered 15 LHCV-related and 13 RHCV-related methylation-gene expression pairs, with five overlapping genes primarily involved in immune regulation. LHCV-specific genes were involved in cellular signalling, and Mendelian randomisation (MR) analyses supported a potential causal association between brain expression of *DIP2C* and increased risk of major depressive disorder. RHCV-specific genes were involved in neuronal differentiation pathways, with MR analyses suggesting that brain-tissue expression of *BAIAP2*, *MACF1*, *SLC16A5*, and *CORO1B* was associated with neuropsychiatric disorders. We also identified sex-specific patterns with hippocampal asymmetry. Notably, baseline methylation at these sites predicted hippocampal atrophy rates, explaining >10% of the variation. Associations with multiple healthy dietary patterns suggest modifiable influences on hippocampal structure.

**Interpretation:**

These findings highlight distinct methylation profiles as potential biomarkers or therapeutic targets for neuropsychiatric and neurodegenerative conditions.

**Funding:**

Institutional funds, 10.13039/501100002347Federal Ministry of Education and Research of Germany, 10.13039/100000957Alzheimer's Association.

**Research in context**

**Evidence before this study**

We conducted a comprehensive search of PubMed for studies investigating the molecular basis of hippocampal volume and asymmetry in human populations (from inception to March 30, 2026. Search terms included “hippocampal volume”, “asymmetry”, “atrophy”, or “subcortical volumes” combined with “DNA methylation”, “epigenetics”, and “omics”. Prior studies mostly focused on overall hippocampal volume rather than hemisphere-specific differences, and few examined asymmetry in relation to molecular markers. These studies included both healthy individuals and those with neuropsychiatric or neurodegenerative conditions, but were generally limited by small sample sizes and a lack of integration across multiple molecular layers to understand the biological functions and pathways of the identified signatures. There lacks large-scale meta-analyses exploring the epigenetic basis of hippocampal asymmetry in healthy adult populations.

**Added value of this study**

This large-scale epigenome-wide meta-analysis aimed to examine DNA methylation patterns associated with left and right hippocampal volumes, and their asymmetry, in a population-based sample of over 8000 adults. We identified distinct methylation sites and gene expression profiles that differ between the left and right hippocampus. Importantly, we also highlighted genes linking brain imaging traits to neuropsychiatric conditions through specific biological pathways. For example, in women, lower methylation at a site linked to reduced *ABT1* expression was associated with greater hippocampal asymmetry and an increased risk for autism. Baseline methylation at identified sites also predicted future hippocampal atrophy, suggesting a role in early structural brain changes. Furthermore, healthy dietary patterns were associated with these signatures, highlighting actionable opportunities to reduce hippocampal atrophy.

**Implications of all the available evidence**

Taken together with existing literature, our findings suggest that the molecular mechanisms shaping hippocampal structure are both hemisphere-specific and influenced by modifiable environmental factors. This provides a foundation for using epigenetic profiles as early biomarkers of brain ageing, neuropsychiatric and neurodegenerative disorders. A better understanding of the asymmetrical molecular architecture of the hippocampus may also help explain why conditions such as schizophrenia, autism, and Alzheimer's disease differ by sex and brain hemisphere. These findings support future research into targeted lifestyle or dietary interventions that may modify epigenetic patterns and reduce the risk or progression of hippocampal atrophy and related brain disorders.

## Introduction

The human hippocampus, and particularly its asymmetry, plays a pivotal role in memory, emotional regulation, and cognitive performance.^1^ Accumulating evidence highlights notable structural and functional asymmetries within the hippocampus.^2^, ^3^, ^4^, ^5^, ^6^, ^7^, ^8^, ^9^ Anatomically, the left hippocampal volume (LHCV) is slightly smaller than the right hippocampal volume (RHCV), and functional lateralisation has been observed in task-related activation patterns.^7^^,^^10^^,^^11^ This asymmetry may reflect complementary specialisation, with the left hippocampus more involved in semantic processing and the right in spatial information processing.^2^^,^^5^ Moreover, neuroimaging studies consistently identify the hippocampus as one of the most profoundly affected grey matter structures in major neuropsychiatric and neurodegenerative disorders, including Alzheimer's disease,^12^ major depressive disorder,^13^ schizophrenia,^14^^,^^15^ attention deficit hyperactivity disorder,^16^ and autism spectrum disorder.^10^ Altered asymmetry patterns in the hippocampus have also been implicated in several of these conditions.^10^^,^^11^^,^^15^ Additionally, ageing appears to affect the left and right hippocampus differently.^17^ Despite these findings, the molecular characteristics underlying the differences in LHCV, RHCV, and hippocampal asymmetry remain largely unknown in the general population. Elucidating these molecular characteristics in healthy adults across a wide age range, including younger and middle-aged individuals, could yield granular, hemisphere-specific insights into brain health and neurodegeneration.

Although genome-wide association studies have discovered several genetic variants associated with LHCV, RHCV, and hippocampal asymmetry,^7^^,^^8^^,^^18^ most of these genetic loci are located in non-coding regions, suggesting a role in transcriptional regulation. A key mechanism of transcriptional regulation is DNA methylation,^19^ which involves the addition of methyl groups to genomic DNA and can be influenced by internal and external factors, including dietary changes.^20^ In addition, DNA methylation levels can affect transcription factor binding, which is essential for fine-tuning gene expression.^21^^,^^22^ Thus, the interplay between genetics, DNA methylation, and gene expression may contribute to the variability in LHCV, RHCV, and hippocampal asymmetry across the adult lifespan.

Indeed, a study of 649 Alzheimer's Disease Neuroimaging Initiative participants, combined with CRISPR-Cas9 (epi)genome-editing in mouse models, revealed a causal relationship between a single nucleotide polymorphism (rs1053218) and hypermethylation of CpG site cg26741686. This epigenetic modification led to increased *ANKRD37* gene expression and a reduction in mean hippocampal volume.^23^ An epigenome-wide meta-analysis including 3337 samples found two CpGs and three DNA methylated regions associated with mean hippocampal volume.^6^^,^^24^ However, previous studies primarily assessed total or mean hippocampal volume from heterogeneous and relatively small samples (n < 3400). Therefore, the methylation signatures of LHCV, RHCV, and hippocampal asymmetry in healthy adults remain underexplored. Moreover, previous studies predominantly relied on the HumanMethylation450 array (HM450K), which measures 450,000 CpGs, or assessed specific CpGs, limiting their capability to detect other relevant CpGs. In our study, several cohorts were profiled using the HumanMethylationEPIC array (HM850K), which provides substantially broader genomic coverage (∼850,000 CpGs).^25^ The inclusion of HM850K data, therefore, increases overall coverage and enhances the potential to identify previously uncharacterised methylation markers related to these features. Importantly, few studies have comprehensively assessed the functional characterisation of these methylation differences at the genetic and transcriptional levels, potentially overlooking key regulatory mechanisms that may not be evident when analysing each molecular layer separately. Additionally, whether these methylation signatures are associated with hippocampal atrophy rates over time or modifiable by lifestyle factors such as diet remains unclear.

Here, we meta-analysed epigenome-wide association studies (EWAS) from six population-based cohorts comprising 8156 samples to identify methylation signatures associated with LHCV, RHCV, and hippocampal asymmetry. To determine whether these associations were specific to the hippocampus or reflected global grey matter changes, we also conducted EWAS for left hemisphere grey matter volume, right hemisphere grey matter volume, and global grey matter asymmetry. Leveraging individual-level omics data from the Rhineland Study participants (n > 2624), we performed integrative cross-omics analyses to elucidate the complex regulatory interactions between genetics, methylation, gene expression, and transcription factor binding sites. These analyses prioritised molecular mechanisms underlying hippocampal structural and functional differences. Finally, we assessed the clinical relevance of the identified methylation markers by investigating their associations with ten dietary patterns and longitudinal changes in LHCV, RHCV, and hippocampal asymmetry.

## Methods

### Study participants

The sample included 8156 participants of European ancestry from six population-based cohort studies: the Rhineland Study, the Study of Health in Pomerania (SHIP-Trend), the Framingham Heart Study (FHS) offspring study, the Lothian Birth Cohort (LBC) 1936, the Leiden Longevity Study (LLS), and the Older Australian Twin Study (OATS). Participants with known dementia, Parkinson's disease, prevalent stroke, intracranial tumours, a history of severe head injury, seizures beginning before the age of 25, epilepsy, or multiple sclerosis at the time of MRI scanning were excluded.

### Ethics

The protocol of the Rhineland Study was approved by the ethics committee of the University of Bonn Medical Faculty (Ref: 338/15). The study protocol of SHIP and SHIP TREND was approved by the medical ethics committee of the University of Greifswald (BB042/19). Ethics permission for FHS and genetic research in FHS was obtained from the Institutional Review Board of Boston University Medical Campus (IRB numbers H-32132, H-26671). Ethical approval for the LBC1936 study was obtained from the Multi-Centre Research Ethics Committee for Scotland (MREC/01/0/56) and the Lothian Research Ethics Committee (LREC/1998/4/183; LREC/2003/2/29). The study protocol of LLS was approved by the ethical committee of the Leiden University Medical Centre before the start of the study (P01.113). Approval for the OATS study was obtained from the ethics committees of the Australian Twin Registry, University of New South Wales, University of Melbourne, Queensland Institute of Medical Research and the South Eastern Sydney and Illawarra Area Health Service (HC17414). Each study obtained written informed consent from all participants in accordance with Declaration of Helsinki and was approved by the appropriate institutional review boards (Table S1).

### Brain image acquisition and segmentation

Brain MRI acquisition was obtained either at the same visit as the DNA methylation assessment or, when not available, at the nearest subsequent visit (Table S1). In each study, MRI scans were performed using standardised procedures. The field strength of the scanners used ranged from 1.5 to 3.0 T and T1, T2 and proton-density-weighted scans were obtained for all participants (Table S2). All studies used fully automated segmentation methods to quantify brain imaging phenotypes (i.e., LHCV, RHCV, LGMV, RGMV and estimated total intracranial volume (eTIV)). Hippocampal asymmetry and global grey matter asymmetry were defined as the differences between left and right volumes divided by their sums.

### DNA methylation profiling

Genomic DNA was extracted from whole blood in each cohort according to standard protocols. Levels of DNA methylation were quantified using the Illumina Infinium MethylationEPIC v1 or Methylation450K BeadChip array. Each cohort performed the quality control for DNA methylation data independently, complying with the agreed quality control (QC) guidelines (Table S3). CpH (ch-annotated) probes passing QC were retained to enable an unbiased epigenome-wide analysis, given their potential relevance to brain-related traits.^26^ The methylation level at each site was represented and analysed as a β-value, defined as the intensity of the methylated signal/(intensity of the unmethylated signal + intensity of the methylated signal + 100). A β-value of 0 represents a completely unmethylated CpG site, and a β-value approaching 1 represents a fully methylated CpG site.

### Statistics

**Fig. 1:**
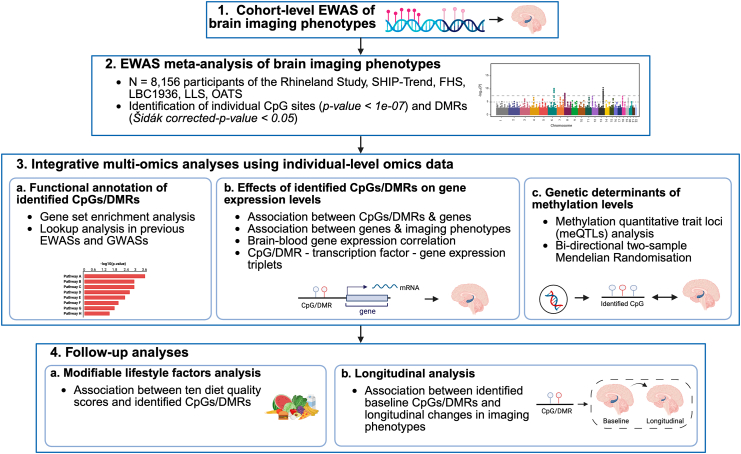
**Workflow for identifying DNA methylation signatures associated with bilateral hippocampal volume and asymmetry**. This workflow presents our analytical overview. The integrative multi-omics analyses and follow-up were performed using individual-level genetics, DNA methylation, gene expression, dietary intake and longitudinal brain imaging data measured in the same participants from the Rhineland Study. Abbreviations: EWAS, epigenome-wide association study; DMR, differentially methylated region; GWAS, genome-wide association study.

Our workflow to identify methylation signatures of LHCV, RHCV, and hippocampal asymmetry is presented in Fig. 1. First, we conducted cohort-level EWAS (step 1) followed by epigenome-wide meta-analysis (step 2), where associations were deemed significant using a stringent epigenome-wide p-value threshold (p < 1 × 10^−7^). Subsequent integrative multi-omics analyses and follow-up analyses were performed using individual-level data from the Rhineland Study and GTEx dataset (steps 3 and 4), for which we applied false discovery rate (FDR) to account for multiple testing. The overview of the datasets used in the analyses is presented in Figure S1.

#### Cohort-level epigenome-wide association analyses (EWAS)

We quantified the association between DNA methylation level (independent variable) and each brain imaging-derived endophenotype (outcome) using multivariable linear regression models in each cohort. β-values were used as the independent variable because they provide direct biological interpretability and ensure consistency across cohorts. Covariates were selected a priori based on known confounders in methylation–phenotype associations, including demographic factors, blood cell composition, and technical sources of variation. Model 1 was adjusted for age, sex (self-reported), estimated or measured blood cell type proportions (%), ancestry-specific genetic principal components (PCs) and technical covariates (i.e., chip, chip position, control probe PCs, and site/scanner). When hippocampal volumes were outcomes, we additionally adjusted for eTIV. Model 2 was additionally adjusted for smoking status and education. We further adjusted for handedness as a sensitivity analysis (Model 3). Covariates assessment in each cohort is detailed in Table S3. To explore whether sex modifies the relationship between DNA methylation and imaging-derived endophenotypes, each cohort also performed sex-stratified analyses.

#### Epigenome-wide meta-analysis

Cohort-level EWAS results were combined using a sample size-weighted random-effect meta-analysis, with association tests based on inverse-variance Wald statistics as implemented in METAL (v.2011-03-25).^27^ We additionally performed sex-stratified meta-analyses and epigenome-wide sex∗methylation interaction analyses. Study-specific results were corrected for genomic inflation during meta-analysis if the genomic inflation factor (λ) was larger than one. An association was considered as epigenome-wide significant if the raw p-value <1.0 × 10^−7^, a sufficiently stringent and commonly used threshold in EWAS studies.^28^^,^^29^ A significant interaction was defined as FDR <0.05 for the interaction term. CpGs on sex chromosomes and cross-reactive CpGs were removed from the results post hoc.

#### Differentially methylated regions analysis

DNA methylation clusters at regions formed by spatially correlated CpGs, namely differentially methylated regions (DMRs), often occur within regulatory regions in the genome and are a powerful means to control gene expression.^30^ To account for this, we performed a DMR analysis to identify a group of methylation sites that collectively influence imaging measures using the Comb-p method.^31^ Comb-p detects regional enrichment of low p-values by applying the Stouffer-Liptak-Kechris test, which combines adjacent CpG-level p-values into a region-level test statistic while accounting for spatial correlation. The significance of each candidate region was then evaluated using the Šidák multiple-testing correction, and a DMR was considered significant if the Šidák-adjusted p-value was <0.05.

#### Gene set enrichment analysis

To explore the biological pathways underlying the effects of methylation on brain imaging measures, we performed gene set enrichment analysis using the missMethyl R package (v.1.42.0).^32^ For each imaging phenotype, CpGs were ranked by their EWAS meta-analysis p-values, and the top 30,000 CpGs were used as the input set, with all tested CpGs included as the background to account for the unequal number of CpGs per gene. To examine the potential influence of methylation direction, the top CpGs were further stratified into hypomethylated and hypermethylated subsets, and pathway analyses were repeated separately for each group.

#### Look-up analysis of identified CpGs in previous EWASs and GWASs

To identify whether the differential CpGs were associated with other traits, we looked up CpGs showing associations with each imaging measure at an epigenome-wide significance level using the EWAS Catalogue (http://ewascatalog.org/). We also performed a look-up of known associations of the mapped gene for each CpG and DMR in previously published GWAS using the GWAS catalogue (https://www.ebi.ac.uk/gwas).

#### Effects of identified CpGs/DMRs on gene expression levels

Effects of identified CpGs on gene expression for mapped genes and *cis*-genes (defined as genes located within ±5 Mb of each CpG, a commonly used window that captures known long-range cis regulatory interactions)^33^^,^^34^ were investigated in 2624 participants of the Rhineland Study for whom DNA methylation and gene expression data were available. Whole blood samples for gene expression sequencing were collected in PAXgene Blood RNA tubes (PreAnalytix/Qiagen; cat. No. 762165) and total RNA was isolated according to the manufacturer's instructions using the PAXgene Blood miRNA Kit and following the automated purification protocol (PreAnalytix/Qiagen; cat. no. 763134). RNA sequencing was performed in paired-end mode (2∗50 cycles) on the NovaSeq6000 platform (Illumina), and the sequencing reads were aligned to the human reference genome GRCh38.p13 provided by Ensembl using STAR (v2.7.1). Additional detailed gene expression quantification and the QC procedure are described in the Supplementary Methods Whole Blood RNA Isolation and Gene Expression Profiling. We investigated the association of identified CpGs with their mapped genes and *cis*-gene expression levels using linear regression models, which provide effect estimates and statistical significance based on t-tests for regression coefficients. Models were adjusted for age, sex, the first ten genetic PCs, and methylation and gene expression batch effects. The analysis of DMRs was performed similarly, except by replacing CpG methylation levels with the median methylation level of all CpGs located within the DMR.^35^ As the DMR analysis already took the nearby regions into account, we restricted the gene expression analysis to the DMR-mapped genes.

#### Association of gene expression with corresponding imaging measures and mediation analysis

For the genes whose expression levels were associated with CpGs/DMRs, we also quantified the association between gene expression levels and the corresponding imaging measures using multivariable linear regression models, which provide effect estimates and statistical significance based on t-tests for regression coefficients. Additionally, we evaluated to what extent gene expression levels mediated the effects of CpGs/DMRs on the respective imaging measures using structural equation modelling (R package laavan v0.6–11). Mediation effects were assessed by estimating the indirect effect, direct effect, and total effect, with statistical significance determined using bootstrap-based tests implemented within the *lavaan* R package (v0.6–11).^36^

For genes whose expression was associated with the identified methylation signatures and also with LHCV/RHCV (defined as key genes), we employed a two-sample Mendelian Randomisation (MR) approach to evaluate whether their expression in brain tissue was potentially causally associated with neurological disorders. Brain expression quantitative trait loci (eQTL) data from GTEx were used as instrumental variables, and causal effects were primarily estimated using the inverse-variance weighted (IVW) method, with statistical significance evaluated via Wald tests. When only a single genetic instrument was available, the Wald ratio test was used.

#### Tissue specificity

To assess the relevance of blood-derived methylation signals to brain-related processes, we evaluated (i) blood–brain CpG methylation correlations using the IMAGE-CpG EPIC resource,^37^ (ii) gene expression correlations between blood and hippocampal tissue using GTEx,^38^ and (iii) brain-tissue functional relevance through two-sample MR analyses using brain eQTLs from GTEx.^38^

#### Putative transcription factors and target genes analyses

Transcription factors (TFs) are proteins that bind to DNA to facilitate transcription, and their binding to DNA can be affected by DNA methylation levels.^21^ To better understand the regulatory roles of the identified CpGs/DMRs on gene expression, we next performed an integrative analysis of DNA methylation, TFs, and gene expression data. We prioritised CpG/DMR-TF-target gene triplets in which regulatory activities of the TFs on target gene expression are most likely influenced by methylation using the *MethReg* R package (v.1.14.0).^39^ Briefly, for CpGs located in the promoter region (within ±2 kb around the transcription start sites (TSS)), we assessed the association between CpG methylation with expression levels of the target genes. For CpGs in the distal regions (>2 kb from TSS), we quantified the association between CpG methylation with expression levels of five nearest genes upstream and downstream from the CpG. Additional analysis details are described in the Supplementary Methods MethReg Integrative Analysis.

#### Methylation quantitative trait loci (meQTLs) analysis and bidirectional two-sample MR analysis

We performed GWASs of the identified CpGs in 6723 participants of the Rhineland Study in whom both genetic and methylation data were available to identify meQTLs. Genotyping was performed on the Omni 2.5 Exome Array, while GenomeStudio (v.2.0.5) was used for genotype calling. Quality control was performed through PLINK (v.1.9) by checking for poor genotyping rate (<99%), Hardy-Weinberg disequilibrium (*p*< 1E − 5), poor sample call rate (<95%), abnormal heterozygosity, cryptic relatedness, and sex mismatch. Additional detailed genetics quantification and the QC procedure are described in Supplementary Methods Genomics and Bidirectional Two-sample Mendelian Randomisation.

The GWAS of each identified CpG was adjusted for age, sex, methylation batch effects, smoking status and the first ten genetic PCs to account for population structure. The genetic PCs were derived using PLINK v1.90b6.7. As shown in the scree plot (Figure S2), the first five PCs explain 59.8% of the genetic variance, followed by a plateau from PC5 onward. Consistent with genetic association study conventions, we adjusted for the first ten PCs,^40^ which capture the major axes of population structure. The genome-wide significance level was set at p-value <5e-8. Next, we performed bidirectional two-sample MR analyses to assess potential causal relationships between the identified CpGs and LHCV/RHCV. In the forward MR analysis, genetic proxies for the identified CpGs were used as the exposure, while genetic proxies for LHCV/RHCV served as the outcome. In the reverse MR analysis, genetic proxies for LHCV/RHCV were used as the exposure, with genetic proxies for the identified CpGs as the outcome. For the CpGs, our GWAS summary statistics were used. For LHCV and RHCV, the UK Biobank GWAS summary statistics (n = 39,691 samples) were used.^41^ The IVW method was used as the primary approach for causal inference. Other MR methods, including the weighted median and the MR Egger method, were applied to assess the robustness of the IVW-based MR estimates. The presence of pleiotropy was assessed through the MR Egger intercept test (p-value <0.05). Additional analysis details are described in the Supplementary Methods Genomics and Bidirectional Two-sample Mendelian Randomisation.

#### Association between diet quality scores and identified CpGs/DMRs

As lifestyle factors are one of the main determinants of methylation changes, we assessed the association between ten diet quality scores and the identified methylation signatures in 5768 participants of the Rhineland Study in whom both dietary and methylation data were available. We included the following diet quality scores in the analysis: Mediterranean-style diet score (MDS), Dietary Approaches to Stop Hypertension (DASH), Mediterranean–DASH Intervention for Neurodegenerative Delay (MIND) diet, the Alternate Healthy Eating Index (AHEI), the Nordic diet score, EAT-Lancet, plant-based diets as assessed by Plant-based Diet Indexes (i.e. overall PDI, healthful PDI, and unhealthful PDI) and Dietary Inflammatory Index (DII). Habitual dietary intake was assessed by a self-administered semi-quantitative food frequency questionnaire (FFQ). Further details are provided in the Supplementary Methods Dietary Assessement and Diet Quality Scores.

#### Association of identified baseline CpGs/DMRs with longitudinal change in imaging measures

To investigate whether the identified baseline methylation signatures were associated with longitudinal changes in brain imaging measures, linear mixed-effect models were applied to data from 2892 Rhineland Study participants who had complete baseline methylation and follow-up imaging data. As with the cross-sectional analysis, we adjusted the models for age, sex, batches, and, in the case of hippocampal volume, for eTIV. All linear mixed-effect models were analysed with the nlme R package (v3.1–168). Further details are provided in the Supplementary Methods.

### Role of funders

The funders had no role in the study's design, data collection, data analysis, interpretation, or the writing of the manuscript.

## Results

### Study sample characteristics

Our meta-analysis included 8156 samples from six population-based cohort studies. The sample characteristics are presented in Table 1. The mean age of the participants of the Rhineland Study, SHIP-Trend, FHS and LLS ranged from 50.4 years to 58.5 years, while participants from LBC1936 and OATS were older (LBC1936 mean age: 72.6 years, OATS mean age: 70.5 years). Sex ratios were balanced in all studies. On average, LHCV was slightly smaller than RHCV in all cohorts, and both LHCV and RHCV decreased with increasing age after adjusting for eTIV (Figure S3). The mean value of hippocampal asymmetry ranged from −0.0058 in FHS to −0.0400 in LBC1936, and slightly increased with age (Figure S3).

**Table 1:** Characteristics of the participating cohorts.

Cohort	Rhineland study	SHIP-Trend	FHS	LBC1936	LLS^a^	OATS
Sample size, n	4568	451	2317	560	173	87
Age (years), mean (SD)	54.4 (13.4)	50.4 (13.4)	57.7 (13.1)	72.6 (0.7)	58.5 (6.6)	70.5 (5.4)
Age range (years)	30–92	22–79	25–90	71–74	40–79	65–83
Female, %	57.4	55.5	47.1	46.6	56.7	58.6
Male, %	42.6	44.5	52.9	53.4	43.3	41.4
Current smoker, %	12.6	21.3	8.2	7.7	14.5	10
Higher education level, %	52.3	26.1	50.4	55.3	36.8	32.6
Estimated total intracranial volume (cm^3^), mean (SD)	1550.00 (148.00)	1573.29 (160.01)	1289.19 (132.60)	1451.20 (143.54)	1426.61 (125.43)	1442.510 (171.671)
Left hippocampal volume (cm^3^), mean (SD)	3.84 (0.44)	3.88 (0.42)	3.36 (0.39)	3.07 (0.44)	5.17 (0.59)	3.66 (0.42)
Right hippocampal volume (cm^3^), mean (SD)	4.00 (0.48)	4.04 (0.43)	3.40 (0.38)	3.33 (0.44)	5.29 (0.53)	3.75 (0.44)
Hippocampal asymmetry index, mean (SD)	−0.0195 (0.0276)	−0.0200 (0.0254)	−0.0058 (0.0290)	−0.0400 (0.0400)	−0.0112 (0.0497)	−0.0118 (0.0304)
Left hemispheric grey matter volume (cm^3^), mean (SD)	256.00 (26.60)	257.51 (26.91)	267.65 (28.18)	201.54 (18.77)	–	193.62 (19.13)
Right hemispheric grey matter volume (cm^3^), mean (SD)	257.00 (26.20)	258.51 (27.08)	267.37 (27.88)	202.95 (19.04)	–	195.15 (18.98)
Hemispheric grey matter asymmetry index, mean (SD)	−0.00136 (0.00536)	−0.00196 (0.00458)	0.00047 (0.0077)	−0.0030 (0.0100)	–	−0.0040 (0.0072)
Methylation array type	HM850K	HM850K	HM450K	HM450K	HM450K	HM450K

Abbreviation: SHIP-Trend, the Study of Health in Pomerania; FHS, the Framingham Heart Study; LBC1936, the Lothian Birth Cohort 1936; LLS, the Leiden Longevity Study; OATS, the Older Australian Twin Study; HM850K, Illumina Infinium MethylationEPIC v1 BeadChip array; HM450K, Illumina Infinium Methylation450K BeadChip array; SD, standard deviation.

a Grey matter volumes are not available in LLS.

### Specific methylation signatures were associated with bilateral hippocampal volume and asymmetry

**Fig. 2:**
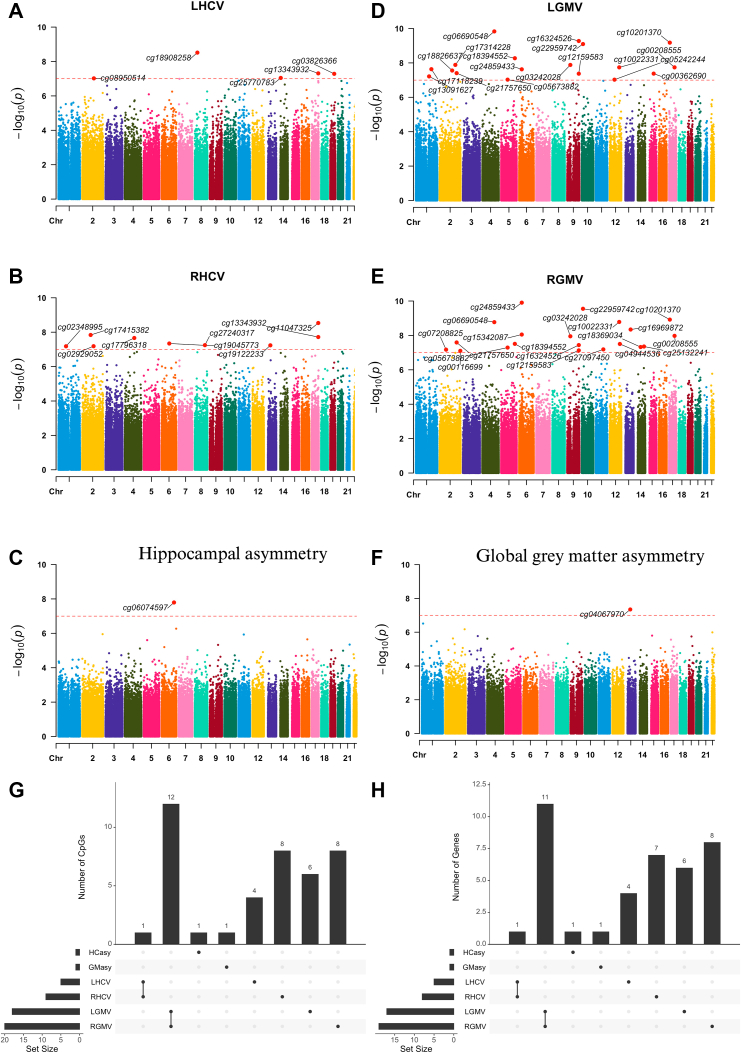
**Manhattan plots of the epigenome-wide meta-analyses of bilateral hippocampal volume and asymmetry**. The sample size for this analysis was 8156. Manhattan plots show methylation signatures of **(A)** left hippocampal volume (LHCV), **(B)** right hippocampal volume (RHCV), **(C)** hippocampal asymmetry, **(D)** left hemisphere grey matter volume (LGMV), **(E)** right hemisphere grey matter volume (RGMV), and **(F)** global grey matter asymmetry. Results were plotted as negative log-transformed p-values (y-axis) across the genome (x-axis). The red horizontal line represents the epigenome-wide significance at 1.0 × 10^−7^, derived from the sample size–weighted random-effects meta-analysis with association tests based on inverse-variance Wald statistics. All the epigenome-wide significant CpGs were annotated. Upset plots show **(G)** the identified CpGs and **(H)** mapped genes overlap among traits. Abbreviations: LHCV and RHCV, left and right hippocampal volumes; LGMV and RGMV, left and right hemisphere grey matter volumes; HCasy, hippocampal asymmetry; GMasy, global grey matter asymmetry.

The meta-analysis (model 2) revealed that five CpGs were associated with LHCV (Fig. 2A), nine CpGs were associated with RHCV (Fig. 2B), and one CpG was associated with hippocampal asymmetry (Fig. 2C & Table S4). Results from model 1 and model 2 were highly consistent (Figure S4), and the top CpGs remained the same after further adjustment for handedness in model 3 (Figure S5), therefore, downstream analyses were based on model 2 results. Intriguingly, minimal overlap was observed in the associated CpGs and their mapped genes among LHCV, RHCV, and hippocampal asymmetry. Moreover, there was no overlap between CpGs associated with hippocampal traits and global grey matter traits (Fig. 2D–H), indicating that the effects were specific to the hippocampus rather than due to global grey matter changes.

Accounting for the joint effect of spatially correlated CpGs clustered in specific genomic regions, we identified 262, 246, and 16 DMRs associated with LHCV, RHCV, and hippocampal asymmetry, respectively (*Stouffer-Liptak-Kechris test*, all Šidák corrected-p-value <0.05, Table S5). CpGs identified from the individual CpG analysis were also detected in the DMR analysis (Table S6). 31.4% of DMRs overlapped between LHCV and RHCV, less than 10% of DMRs overlapped between hippocampal and global grey matter traits, and only two DMRs associated with hippocampal asymmetry were also related to LHCV and/or RHCV (Figure S6).

### *In silico* replication

Previous EWAS analyses identified three CpGs (cg17858098, cg26927218, and cg26741686) and two DMRs associated with mean hippocampal volume.^24^ In our study, these two DMRs (nearest genes: *ALLC and SLC16A3*) were also associated with LHCV/RHCV (Table S7). In addition, we found that higher levels of cg17858098 methylation tended to be associated with larger RHCV, but smaller LHCV (Table S7). Although cg26927218 was not present in our study, previous EWAS found it to be associated with *BAIAP2* gene expression. Our study showed that higher methylation levels of two RHCV-related CpGs (cg11047325 and cg13343932) were associated with higher *BAIAP2* gene expression.

### Gene set enrichment analysis for the identified methylation signatures

These identified methylation signatures largely converged on pathways including, but not limited to, nervous system development, neuron differentiation, generation of neurons, and neuronal morphogenesis (Figures S7 & S8 & Tables S8 & S9). Moreover, these distinct CpGs/mapped genes were involved in trait-specific biological pathways (Figures S8 & S9). LHCV- and RHCV-related DMR-mapped genes have been linked to hippocampal volume in previous GWAS analyses, confirming their (epi)genetic function in the hippocampus. These DMR-mapped genes have also been linked to a wide range of neurodegenerative and psychiatric disorders, as well as to CSF Aβ_1-42_ levels, t-tau/Aβ_1-42_ ratio, and p-tau181p levels (Table S10).

In addition, among the top 30,000 CpGs ranked by their EWAS meta-analysis p-values, there were slightly more hypomethylated than hypermethylated sites for each trait (LHCV: 15,523 hypo vs. 14,555 hyper; RHCV: 15,140 hypo vs. 14,860 hyper; hippocampal asymmetry: 15,445 hypo vs. 14,555 hyper). The enriched pathways differed between hypomethylated and hypermethylated CpGs. For example, among LHCV-associated CpGs, hypermethylated loci were involved in T-cell receptor signalling, phosphatidylinositol signalling, and Th17 cell differentiation, whereas hypomethylated loci were involved in axon guidance, parathyroid hormone synthesis, and cholinergic synapse pathways (Figure S10).

### Associations of identified methylation markers with gene expression and corresponding phenotypes

**Fig. 3:**
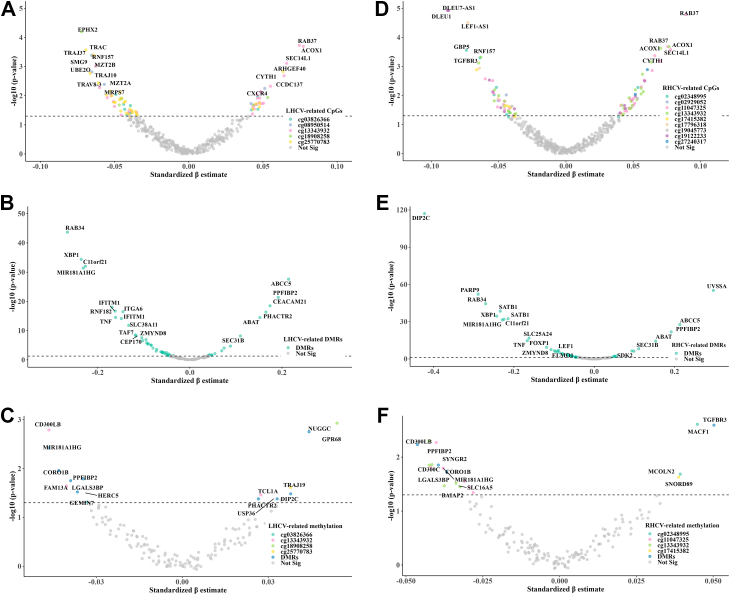
**The associations between identified methylation signatures, gene expression, and bilateral hippocampal volume**. The sample size for this analysis was 2624. Volcano plots show the associations between **(A)** LHCV-related CpGs with gene expression (mRNA), **(B)** LHCV-related DMRs with mRNA, **(C)** significant mRNAs from A&B with LHCV; **(D)** RHCV-related CpGs with mRNA, **(E)** RHCV-related DMRs with mRNA, **(F)** significant mRNAs from D&E with RHCV. Each dot represents a gene. Coloured dots indicate mRNAs significantly associated (above the horizontal dashed line) with either methylation signatures or hippocampal volume (multivariable linear regression t-statistic), with colour denoting the corresponding methylation feature (CpG or DMR). Abbreviations: LHCV and RHCV, left and right hippocampal volumes; DMR, differentially methylated region.

All five LHCV-related CpGs showed associations with their *cis*-gene expressions, resulting in 82 significant CpG-gene expression pairs (Fig. 3A & Table S11). Additionally, 55 of the 134 LHCV-related DMRs were associated with their gene expression (Fig. 3B & Table S12). Among them, the expression levels of 15 genes were also associated with LHCV (Fig. 3C & Tables S13 & S14). The DMRs encompassing the top CpGs showed slightly stronger associations with gene expression compared to individual CpGs. Similarly, all nine RHCV-related CpGs showed associations with the expression levels of their *cis*-genes, yielding 104 significant CpG-gene expression pairs (Fig. 3D & Table S11), and 46 of the 115 RHCV-related DMRs were associated with their gene expressions (Fig. 3E & Table S12). Among them, the expression levels of 13 genes were also associated with RHCV (Fig. 3F & Tables S13 & S14). Hippocampal asymmetry-related cg06074597 and DMRs were associated with the expression of seven genes (Tables S12–S14). However, none of these gene expression levels were significantly associated with hippocampal asymmetry. Most methylation-gene expression associations were negative (i.e., higher methylation corresponding to lower gene expression, Fig. 3), particularly for DMRs located in regulatory regions such as the TSS, 5′UTR, and CDS (Table S15). Positive associations were also observed, mainly for DMRs in intronic or intergenic regions, where methylation–expression relationships are more complex (Table S15).

Further mediation analyses revealed nominally significant mediation of the associations between the DMRs chr11.7597983.7598302 and chr1.39620300.39620625 with RHCV through the gene expression of *PPF1BP2* (25.7% mediation) and *MACF1* (16.9% mediation) (bootstrap-based tests, p-values < 0.05, but FDR >0.05, Table S16). The mediation effects for other CpG/DMR-gene-trait pairs were not statistically significant, likely due to the relatively smaller sample size (n = 1800 participants with available methylation, gene expression, and brain MRI data).

### Sex-specific patterns in methylation–gene expression associations related to hippocampal asymmetry

**Fig. 4:**
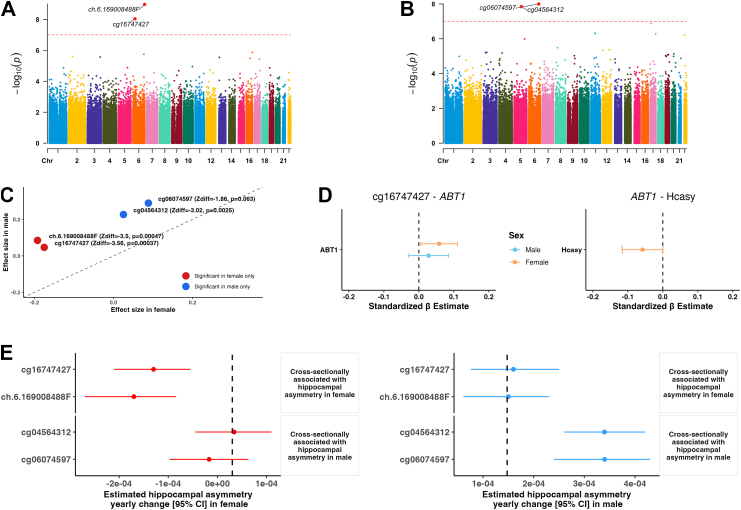
**Sex difference in single CpG association with hippocampal asymmetry**. The sample size for this analysis was 4218 (females) and 3938 (males). Manhattan plots show epigenome-wide associations between DNA methylation and hippocampal asymmetry in **(A)** female and **(B)** male. The y-axis represents the −log_10_-transformed p-values, plotted across the genome (x-axis). The red horizontal line indicates the epigenome-wide significance threshold (p = 1.0 × 10^−7^), derived from the sample size–weighted random-effects meta-analysis with association tests based on inverse-variance Wald statistics. Epigenome-wide significant CpGs are annotated. **(C)** Beta–beta plots depict effect sizes for the four significant CpGs in both female and male. Z-tests were used to evaluate sex differences in effect estimates. **(D)** Forest plots show associations between sex-differentiated CpGs, gene expression levels, and hippocampal asymmetry (HCasy) in female and male. Dots represent mean effect estimates, and horizontal lines indicate 95% confidence intervals (CIs). **(E)** Forest plots show sex-stratified associations between CpG methylation and longitudinal change in hippocampal asymmetry in female and male. Dots represent mean effect estimates, and horizontal lines indicate 95% confidence intervals (CIs). Abbreviations: HCasy, hippocampal asymmetry; CI, confidence interval.

In formal sex × methylation interaction tests in the full sample for LHCV, RHCV, and hippocampal asymmetry, no CpG reached statistical significance after FDR correction for any trait, indicating no robust evidence that methylation–trait associations differ by sex at an epigenome-wide level (multivariable linear regression t-statistic, all FDR for interaction terms >0.05, top hits shown in Table S17). Nevertheless, four CpGs showed differing associations with hippocampal asymmetry in men and women in the sex-stratified analyses (Fig. 4A–C, Table S18). Specifically, in women, higher methylation levels of cg16747427 and ch.6.169008488F were associated with less hippocampal asymmetry cross-sectionally (Fig. 4A). Cg16747427 was associated with 11 *cis*-gene expression levels, among which lower expression *of* the *ABT1* gene was associated with increased hippocampal asymmetry in women (Fig. 4D & Table S19). In men, higher methylation at cg04564312 and cg06074597 was associated with more hippocampal asymmetry cross-sectionally (Fig. 4B), and higher cg04564312 levels were associated with lower *RGMB* gene expression (Table S19).

Importantly, in women, higher baseline methylation at cg16747427 and ch.6.169008488F was significantly associated with decreased hippocampal asymmetry over time (Fig. 4E). These two CpGs together explained 4.4% of the variation in hippocampal asymmetry change rates in women (Table S20). In men, higher baseline methylation at cg04564312 and cg06074597 was associated with increased hippocampal asymmetry over time. These two CpGs together explained 30% of the variation in hippocampal asymmetry change rates in men (Fig. 4E & Table S20).

### Functional divergence of left and right hippocampus key genes and their association with neuropsychiatric disorders

**Fig. 5:**
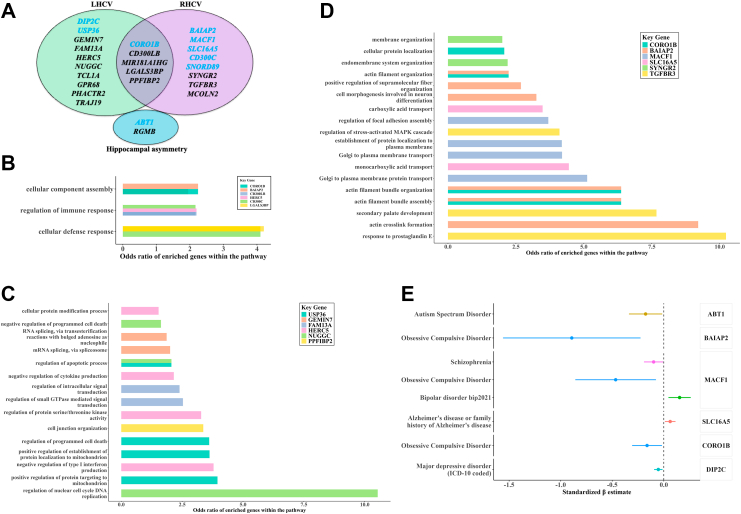
**Functional characterisation of key genes associated with bilateral hippocampal volume and asymmetry**. The sample size for this analysis was 2624. **(A)** A Venn diagram shows the overlap of identified mRNAs among traits. Bar plots show **(B)** shared Gene Ontology (GO) biological processes across traits, **(C)** GO biological processes specifically implicated in left hippocampal volume (LHCV), and **(D)** GO biological processes specifically implicated in right hippocampal volume (RHCV). **(E)** Forest plot shows effect estimates of brain mRNA expression on neurological disorders based on Mendelian Randomisation analysis. Dots represent mean effect estimates, and horizontal lines indicate 95% confidence intervals (CIs). Abbreviations: LHCV, left hippocampal volume; RHCV, right hippocampal volume; MR, Mendelian Randomisation; GO, Gene Ontology.

Of the 25 genes whose expression was associated with the identified methylation signatures and also with LHCV/RHCV, only five were associated with both LHCV and RHCV (Fig. 5A). These overlapping genes were primarily involved in the regulation of immune response, cellular defence mechanisms, and cellular component assembly (Fig. 5B). Pathway analysis revealed that LHCV-associated genes were mainly involved in cellular signalling and protein biogenesis pathways (Fig. 5C), whereas RHCV-associated genes were more linked to neuronal differentiation and organisation processes (Fig. 5D).

Although blood–brain CpG methylation correlations for our identified sites were modest (ranging from −0.03 to 0.33; Table S21), comparative expression analysis using GTEx showed a Pearson correlation of 0.56 between the gene expression levels of these 25 genes in blood and hippocampal tissue (Table S22), supporting partial cross-tissue consistency. Notably, MR analysis using brain eQTLs suggested that the expression of several identified genes in brain tissues may be causally associated with neuropsychiatric disorders (Fig. 5E & Figure S11). Specifically, altered expression of *BAIAP2*, *MACF1*, and *SLC16A5* in brain tissue was associated with obsessive-compulsive disorder, schizophrenia, and Alzheimer's disease (Fig. 5E; all FDR <0.05, all F-statistic >20, Table S23). There was no evidence for heterogeneity (Cochran's Q test, all p-value >0. 5). Moreover, reduced expression of *ABT1*, a gene associated with increased hippocampal asymmetry specifically in women, showed evidence of a potential causal relationship with increased risk of autism spectrum disorder (IVW beta = −0.177, se = 0.082, FDR = 0.036; Fig. 5E), a condition known to exhibit pronounced sex-differnces in both prevalence and clinical presentation.^42^

### Hippocampus-related methylation signatures were associated with putative transcription factors and target genes

We found that the majority of the identified methylation signatures were located in the promoter regions and the gene body (Figure S12) and were enriched in gene regulatory elements (Table S24), supporting their dynamic interaction with transcription factors (TFs) in gene expression regulation. Our integrative analysis of DNA methylation, TF binding, and gene expression suggested that methylation at cg25770783 may attenuate the activation of the transcription factor FOXO4 on its target gene *DHRS1* (Figure S13 &Table S25). For example, among participants in the lowest quartile of cg25770783 methylation levels, higher FOXO4 activity was associated with slightly higher *DHRS1* expression. In contrast, among participants in the highest quartile of cg25770783 methylation, there seemed to be no association between FOXO4 activity and *DHRS1* expression. Similarly, several additional TFs appeared to interact with identified DMRs to modestly influence target gene expression (Table S25). For instance, methylation at DMR chr1:174,843,523–174,843,971 appeared to enhance the repression of the transcription factor ZNF354C on its target gene *GPR52*, while methylation at DMR chr15:31,685,635–31,685,823 appeared to attenuate the repression of ZNF354C on its target gene *OTUD7A* (Figure S13). While these interaction patterns were statistically significant, their magnitudes were small, which is to be expected for molecular regulatory effects. These findings should therefore be considered hypothesis-generating and warrant further mechanistic and experimental investigation.

### meQTLs analysis and bidirectional two-sample Mendelian Randomisation associations

Our GWAS analyses identified 595, 228, and 154 genome-wide significant SNPs associated with three LHCV-related CpGs (Figure S14A & Table S26). For the nine RHCV-related CpGs, we identified 3188 genome-wide significant SNP-CpG pairs (Figure S14B & Table S26). After linkage disequilibrium (LD)-clumping, the independent SNPs for each CpG were used as genetic instrumental variables in the subsequent MR analyses (Table S27).

The bidirectional two-sample MR suggested a potential causal relationship between higher methylation at cg19045773 and smaller RHCV (IVW beta = −0.945, se = 0.417, p-value = 0.023, F-statistic = 376.57, Figure S15A & Table S27). The effect size and direction were consistent across MR models, with no evidence of heterogeneity (Cochran's Q test, p-value = 0.701) or horizontal pleiotropy (MR Egger regression intercept p-value = 0.399, Table S27). Additionally, methylation at cg19045773 was associated with expression levels of the *cis*-genes *RRAGD* and *ANKRD6* (Figure S15B). We also found evidence suggesting that higher methylation at cg02929052 was causally associated with larger RHCV (IVW beta = 2.063, SE = 0.956, p-value = 0.03, F-statistic = 222.13, Figure S15A & Table S27). There was no evidence for heterogeneity (Table S27). In addition, higher cg02929052 methylation was associated with lower *AMMECR1L* and *UGGT1* gene expression (Figure S15B).

### The association between diet quality scores and identified methylation signatures

**Fig. 6:**
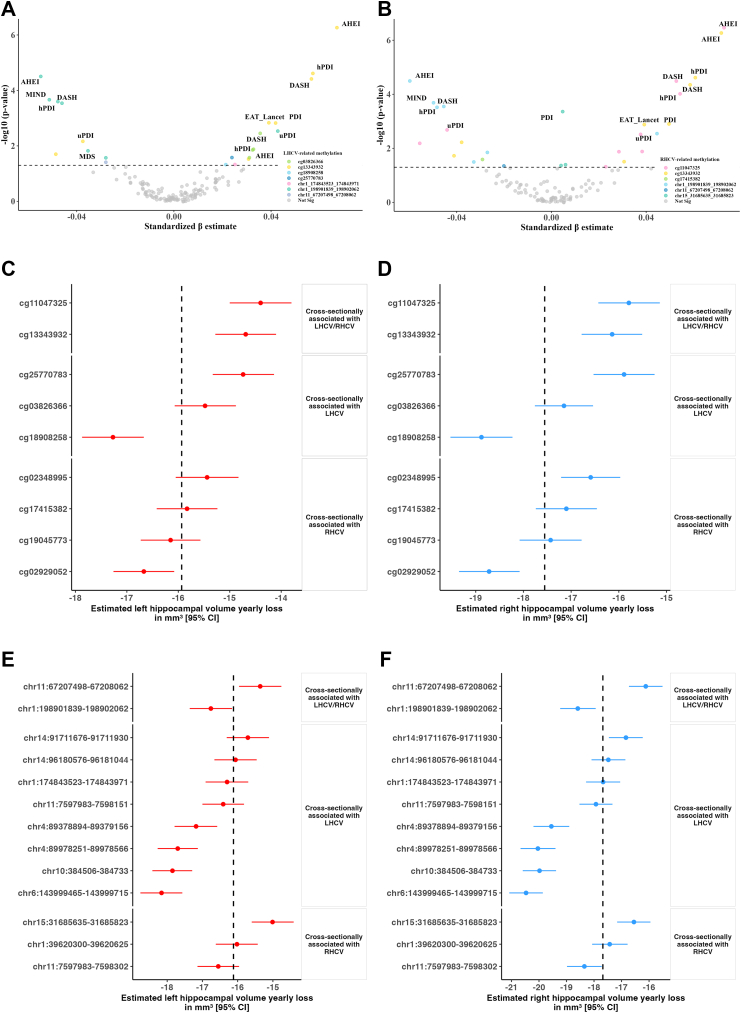
**Associations between dietary patterns, identified methylation signatures, and longitudinal change in imaging measures**. Volcano plots show the associations between ten dietary patterns and **(A)** LHCV-related methylation signatures, **(B)** RHCV-related methylation signatures. The sample size for the dietary analysis (A&B) was 5768. Annotated dots indicate dietary patterns significantly associated with methylation signatures (above the horizontal dashed line), with colour denoting the corresponding methylation feature (CpG or DMR). Forest plots show the association between identified baseline CpG with **(C)** left and **(D)** right hippocampal volume loss, and of the identified baseline DMR with **(E)** left and **(F)** right hippocampal volume loss. The sample size for the longitudinal analysis (C–F) was 2892. The dot represents the mean annual change rate in imaging measures per standard deviation increase in baseline methylation, while the horizontal line indicates the 95% confidence intervals (CIs). The dashed line in each plot indicates the yearly change rate of each measure when CpG expression was equal to the population mean, averaged across all CpGs included in the plot. To facilitate plotting, different axis scales have been used for hippocampal volume changes. Abbreviations: MDS, Mediterranean Diet Score; DASH, Dietary Approaches to Stop Hypertension; MIND, Mediterranean-DASH Intervention for Neurodegenerative Delay; AHEI, Alternate Healthy Eating Index score; PDI, Plant-based Diet Index; hPDI, Healthful Plant-based Diet Index; uPDI, Unhealthful Plant-based Diet Index; DII, Dietary Inflammatory Index; LHCV and RHCV, left and right hippocampal volumes; DMR, differentially methylated region; CI, confidence interval.

We observed that higher adherence to healthy dietary patterns, such as AHEI, hPDI, DASH, EAT-Lancet and Nordic diets, was consistently associated with higher methylation levels at several CpGs, specifically cg133343932 and cg11047325. Conversely, adherence to unhealthy dietary patterns (e.g., DII and unhealthful PDI) was linked to lower methylation levels at the same CpGs (Fig. 6A and B). Similarly, we found that greater adherence to multiple dietary patterns was associated with lower methylation levels at LHCV/RHCV-related DMRs, particularly those mapped to *MIR181A1HG (chr1:198901839-198902062)* or *CORO1B (chr11:67207498-67208062)* (Fig. 6A and B).

### Association of identified baseline methylation signatures with longitudinal changes in imaging measures

Among the CpGs cross-sectionally associated with LHCV, higher baseline methylation at cg13343932 and cg25770783 was significantly associated with slower left hippocampal volume loss over time, while higher baseline methylation at cg18908258 was associated with faster left hippocampal volume loss over time (Fig. 6C). For instance, the average annual LHCV change rate was −15.83 mm^3^ in the linear mixed model for cg25770783. However, when baseline cg25770783 methylation was one standard deviation higher, the estimated average annual change rate improved to −14.74 mm^3^, reflecting a 7.5% reduction (1.1 mm^3^) in yearly left hippocampal volume loss (Table S28). Notably, these three CpGs together explained 15.6% of the variation in left hippocampal volume loss rates. Similarly, among the six CpGs cross-sectionally associated with RHCV, higher baseline methylation at cg11047325, cg13343932 and cg02348995 was significantly associated with slower right hippocampal volume loss over time, whereas higher baseline methylation at cg02929052 was associated with faster right hippocampal volume loss over time (Fig. 6D &Table S28). These four CpGs together explained 10.9% of the variation in right hippocampal volume loss rates.

At the DMR level, among the DMRs cross-sectionally associated with LHCV, higher baseline methylation at five DMRs was significantly associated with slower left hippocampal volume loss over time, and higher baseline methylation at one DMR was associated with faster left hippocampal volume loss over time (Fig. 6E & Table S29). For RHCV, higher baseline methylation at two DMRs was associated with slower right hippocampal volume loss over time, and higher baseline methylation at another two DMRs was significantly associated with faster right hippocampal volume loss over time (Fig. 6F & Table S29).

## Discussion

By leveraging EWAS across several population-based cohorts, we identified distinct DNA methylation patterns in blood that were associated with left and right hippocampal volumes, as well as hippocampal asymmetry, in healthy adults. Our integrative approach, combining methylation, transcriptomic data, and Mendelian randomisation, revealed biologically divergent molecular signatures for left and right hippocampal volume. Genes associated with LHCV were involved in pathways related to cellular signalling and protein biogenesis, and their brain tissue expression levels (e.g., *DIP2C and COR1B*) showed evidence of causal relationships with major depressive disorder and obsessive compulsive disorder. In contrast, RHCV-associated genes were implicated in neuronal differentiation and organisation, with MR analyses linking their expression (*BAIAP2*, *MACF1*, *SLC16A5*, and *CORO1B*) to a range of neuropsychiatric conditions, including autism spectrum disorder, bipolar disorder, obsessive compulsive disorder, and schizophrenia. We also uncovered sex-specific epigenetic patterns with hippocampal asymmetry. Importantly, various dietary patterns were associated with methylation levels at these loci, and baseline methylation signatures at these loci predicted longitudinal changes in hippocampal volume and asymmetry, collectively explaining >10% of the variation in bilateral hippocampal atrophy change rates.

Our findings reveal sex-specific epigenetic patterns with hippocampal asymmetry and highlight potential underlying molecular mechanisms. Specifically, in women, two CpG sites, cg16747427 and ch.6.169008488F, were consistently associated with hippocampal asymmetry both cross-sectionally and longitudinally. Notably, cg16747427 was linked to the expression of *ABT1* (activator of basal transcription 1), a gene involved in transcriptional regulation and potentially in the orchestration of gene networks critical for neurodevelopment and neuroplasticity. Mendelian randomisation analysis further suggested a causal relationship between reduced *ABT1* expression and increased risk of autism spectrum disorder, a condition characterised by marked sex differences in both prevalence and clinical manifestation. This association is particularly compelling given prior links between *ABT1* and intelligence,^43^ cognitive ability^44^ and depression.^45^ Conversely, in men, two distinct CpG sites, cg04564312 and cg06074597, were identified. cg04564312 was associated with the expression of the *RGMB* (repulsive guidance molecule B) gene, which is involved in axonal guidance and neuronal connectivity processes^46^ that may contribute to hippocampal lateralisation. These findings suggest that sex-specific epigenetic regulation of key neurodevelopmental genes, such as *ABT1* and *RGMB* in hippocampal asymmetry across the lifespan, with implications for understanding the biological basis of sex-differential risk in neuropsychiatric and neurodevelopmental disorders.

Our functional omics analyses revealed potential common and specific molecular mechanisms through which gene methylation influences bilateral hippocampal atrophy. Genes associated with LHCV were primarily involved in pathways related to cellular signalling and protein biogenesis, suggesting molecular pathways that support cellular growth, synaptic maintenance, and metabolic regulation within the hippocampus. Among these, *USP36* encodes a nucleolar deubiquitinating enzyme implicated in ribosome biogenesis and chromatin remodelling,^47^ processes essential for neuronal transcriptional activity and synaptic plasticity. *DIP2C*, on the other hand, is thought to regulate epigenetic and transcriptional control during neurodevelopment and has been associated with neurocognitive performance.^48^ MR analyses indicated that altered expression of *DIP2C* in brain tissue was associated with major depressive disorder, underscoring the potential importance of protein homoeostasis and gene regulation in the left hippocampus for maintaining mental health. In contrast, genes uniquely associated with RHCV were enriched for pathways involved in neuronal differentiation and organisation, reflecting the right hippocampus's role in spatial memory and cognitive integration. *BAIAP2* encodes a synaptic scaffolding protein critical for dendritic spine morphology and has been strongly linked to autism and other neurodevelopmental disorders.^49^^,^^50^ *MACF1* (microtubule actin crosslinking factor 1) plays a role in cytoskeletal dynamics, essential for neuronal migration and connectivity.^51^ *SLC16A5* encodes a monocarboxylate transporter involved in cellular energy homoeostasis, and its expression has been associated with Alzheimer's disease.^52^ *SNORD89* is a small nucleolar RNA that may contribute to RNA editing and neuronal transcript stability.^53^ Expression levels of these genes were found to be causally associated with a broad spectrum of neuropsychiatric conditions, including autism spectrum disorder, bipolar disorder, obsessive compulsive disorder, schizophrenia, and Alzheimer's disease, highlighting the right hippocampus as a critical structure for molecular pathways governing neural architecture, connectivity, and disease vulnerability.

In addition, *CORO1B, CD300LB, MIR181A1HG, LGALS3BP, and PPFIBP2* were significantly associated with both left and right hippocampal volume, and these overlapping genes are implicated in immune regulation. Adherence to healthy dietary patterns was strongly associated with these same methylation signatures, suggesting that dietary intake could modulate the rate of hippocampal atrophy through its effects on DNA methylation. The *CORO1B* (Coronin 1B) gene is essential for regulating the actin cytoskeleton,^54^ which is crucial for neuronal structure and synaptic function. The CD300 family of molecules regulates a diverse array of immune cell processes. Among these, *CD300LB* (also known as *TREM5* or *CLM7*) is an activating receptor of the immunoglobulin (Ig) superfamily, predominantly expressed on myeloid cells. *CD300LB* plays a critical role in modulating immune responses, frequently by interacting with lipid ligands and other cellular components.^55^ Nutritional patterns associated with reduced inflammation and enhanced immune resilience, such as the EAT-Lancet, AHEI, and Nordic diets, may influence the regulation of *CD300LB*. These diets might enhance the receptor's capacity to maintain a balanced immune response, contributing to the protection of neural tissue from excessive inflammation. Conversely, diets high in processed foods and pro-inflammatory components could dysregulate *CD300LB* activity, potentially exacerbating immune-mediated damage in the central nervous system.^56^ The *MIR181A1HG* gene encodes microRNA-181a, which has been linked to cognitive function and has been proposed as a potential target against cognitive decline,^57^^,^^58^ Prior dietary intervention studies suggest that diet can modulate microRNA expression, including miR-181a, potentially through plant-based dietary patterns rich in anti-inflammatory and antioxidant components (i.e., fruits, vegetables, nuts, etc.).^59^, ^60^, ^61^ These dietary ingredients have been shown to influence inflammation-related epigenetic pathways, supporting biological plausibility for the diet–methylation associations observed in our study.^62^^,^^63^ For example, dietary interventions have been associated with methylation changes in inflammatory and metabolic genes: IL6 methylation increased following energy restriction in obese women and decreased after bariatric surgery, while improved response to an 8-week energy-restricted diet was linked to lower methylation of LEP and TNF-α.^60^^,^^61^

We found evidence for a causal association of cg19045773 with RHCV, with effects on *ANKRD6* and *RRAGD* expression. *ANKRD6*, expressed in neuronal proliferation zones, is involved in brain development^64^ and has been linked to sex-specific functional connectivity changes in depression.^65^ This aligns with prior findings, which found a causal relationship between cg26741686 hypermethylation and higher *ANKRD37* gene expression in reduced mean hippocampal volume,^23^ with *ANKRD37* involved in hypoxia, which facilitates the pathogenesis of late-onset Alzheimer's disease by accelerating Aβ accumulation, increasing tau hyperphosphorylation, impairing the blood–brain barrier, and promoting neuronal degeneration.^66^ Another RHCV-suggested causal CpG site, cg02929052, was associated with lower *UGGT1* and *AMMECR1L* gene expression. Studies have found that *UGGT1* and other N-glycan modification enzymes are colocalised with Aβ plaques and neurofibrillary tangles in AD brains, suggesting a role in driving glycoprotein remodelling and AD pathogenesis.^67^

Our integrative analysis of methylation, transcription factors and gene expression revealed several TFs that interact with LHCV/RHCV-associated CpGs to co-regulate target gene expression. For instance, we identified that methylation at cg25770783 and the transcription factor FOXO4 jointly regulate *DHRS1* gene expression related to LHCV. *DHRS1* encodes a member of the short-chain dehydrogenases/reductases (SDR) family, which catalyses the reduction of steroids, participating in steroid and/or xenobiotic metabolism.^68^ FOXO4 is one of the fundamental anti-stress signalling molecules. In humans, the FOXO family and their downstream effectors are thought to be critical in reducing inflammation and are a potential nutraceutical approach to healthy ageing and lifespan extension.^69^ In addition, we found that two DMRs and TF ZNF354C jointly regulate *GPR52 and OTUD7A* gene expression related to hippocampal volume. Previous studies have found that ZNF354C was highly expressed in the brain, including the prefrontal cortex, hippocampus and amygdala, and its high expression in the hippocampus has been linked to the onset of depression.^70^ *GPR52* plays important roles in signal transduction, and may impact locomotor activity through modulation of dopamine, NMDA and ADORA2A-induced locomotor activity.^71^ OTUD7A protein acts on TNF receptor-associated factor 6 to control nuclear factor kappa B expression, and an is an emerging independent psychiatric and neurodevelopmental disorders risk gene.^72^^,^^73^

Our study has both strengths and limitations. A key strength of this study is the integration of multi-omics data with imaging-derived measures of hippocampal structure across large, well-characterised cohorts, enabling a robust and comprehensive examination of molecular correlates of hippocampal volume, asymmetry, and atrophy. By combining cohort-level EWAS, meta-analysis, and follow-up multi-omics analyses, the study provides a framework for examining how molecular variation may relate to brain morphology and potentially to neurodevelopmental and neuropsychiatric conditions. Additionally, sex-stratified and lifestyle-related analyses offer insight into possible sex-, hemisphere-, and lifestyle-specific patterns that can guide future hypothesis-driven research. Despite these strengths, several limitations should be considered. First, our omics data were derived from blood samples, which may not comprehensively capture brain-specific methylation patterns. To address this, we extrapolated our findings by assessing the expression of the identified genes across brain tissues and directly compared DNA methylation patterns between blood and brain. Second, each cohort used a different brain imaging segmentation algorithm, which could introduce bias. Nevertheless, the quantified brain volumetric measures remained comparable across cohorts, supporting the robustness of our findings. In a subset of FHS participants, DNA methylation preceded MRI by several years, which may have introduced non-differential measurement error and likely biased associations toward the null. Third, FFQ-based dietary assessments are subject to recall and reporting bias, and prospective dietary intervention studies will be needed to investigate whether dietary patterns directly influence the methylation signatures identified here. In addition, our findings are based predominantly on individuals of European ancestry, and their generalisability to other ancestral groups remains to be investigated.

Together, these findings highlight that hippocampal volume and asymmetry are shaped by distinct molecular mechanisms with relevance to brain health, sex-specific vulnerability, and psychiatric disease risk, underscoring the critical role of epigenetic regulation in brain structure. The methylation signatures identified in this study may serve as potential blood-based biomarkers or therapeutic targets for age- or neurodegeneration-related hippocampal atrophy.

## Contributors

DL, NAA and MMBB conceptualised the study and contributed to the methodological design. DL, VT, JFT, RW, MAI, AT, KW, RFH, DV, NJA, SE, NAR, WW, and KAM performed formal analysis. DL wrote the original draft of the manuscript, and KM, VT, and NAA edited the first draft. Cohort principal investigators (MB, HV, RB, JMW, PSS, PES, SRC, HJG, QY, and MMBB) contributed to data curation and accessed and verified the underlying data. All authors critically reviewed the manuscript and approved the final version.

## Data sharing statement

The data supporting the findings of this study are included in the manuscript and its supplementary materials. The complete EWAS summary statistics and analysis scripts are available via Zenodo (https://doi.org/10.5281/zenodo.18926492). Individual-level dataset is not openly accessible due to data protection regulations and participant privacy constraints. Access to these data can be granted to qualified researchers in accordance with each cohort's Data Use and Access Policy. Access requests should be directed to the relevant cohort data access committee.

## Declaration of interests

N. Ahmad Aziz has received research funding from the H2020 European Research Council (H2020 Excellent Science, European Research Council, grant 101041677) and from the InVirtuo 4.0 Grant, for which he serves as co-principal investigator on a project funded by the Ministry of Culture and Science of the State of North Rhine–Westphalia. He is also a member of the European Huntington's Disease Network, serves on the Advisory Board of the International Society for Neurodegenerative Diseases, and is a member of the Executive Committee of the European Huntington's Disease Network. Alexander Teumer has received research funding from the DFG. Hans J. Grabe has received research support from the DFG and the German Ministry for Education and Research, as well as travel grants and speaker honoraria from Neuraxpharm, Servier, Indorsia, and Janssen Cilag. Joanna M. Wardlaw chairs the European Stroke Organisation guidelines group in small vessel disease; this role has no financial implications. Robert Hillary has received support from Illumina as part of an internal grant. Perminder S. Sachdev has received research funding from the National Health and Medical Research Council of Australia (APP1169489, paid to institution) and from the U.S. National Institutes of Health (grants 1RF1AG057531–01 and 2R01AG057531–02A1, paid to institution). He has also received personal fees from Alkem Labs for a lecture in the Frontiers of Psychiatry 2023 seminar (Mumbai, India); from Biogen Australia for participation in medical advisory committees in 2020 and 2021; from Roche Australia for participation in a medical advisory committee in 2022; and from Eli Lilly and Novo Nordisk for participation in expert advisory panels in 2025. He is an unpaid Executive Board member of the International Neuropsychiatric Association and an unpaid member of the Planning Committee of the World Psychiatric Association. All other authors declare no conflicts of interest.
