# Supplementary material for: DNA methylation signatures of bilateral hippocampal volume, asymmetry and atrophy: a cross-omics analysis in the general population
Source: eBioMedicine. 2026 May 11;128:106289. doi: 10.1016/j.ebiom.2026.106289 (PMC13191633; doi:10.1016/j.ebiom.2026.106289)
Supplement: Supplementary Methods [file mmc1.docx]

**DNA methylation signatures of bilateral hippocampal volume, asymmetry and atrophy: a cross-omics analysis in the general population**

**D. Liu et al.**

**Supplementary Methods**

**Whole Blood RNA Isolation and Gene Expression Profiling**

We extracted total RNA from blood. Blood was collected in PAXgene Blood RNA tubes (PreAnalytix/Qiagen; cat. No. 762165) and processed according to the manufacturer’s guidelines. PAXgene tubes were thawed and incubated at room temperature to increase RNA yields. Total RNA was isolated according to the manufacturer’s instructions using PAXgene Blood miRNA Kit (PreAnalytix/Qiagen; cat. no. 763134) and following the automated purification protocol. RNA integrity and quantity were evaluated using the Tapestation 4200 instrument (Agilent). We used 750 ng total RNA to generate NGS libraries using the TruSeq stranded total RNA kit (Illumina; cat. no. 20020613) following the manufacturer’s instructions with Ribo-Zero Globin reduction. We quantified the libraries via Qubit HS dsDNA assay (Invitrogen; cat. no. Q32851) and clustered at 250 pM final clustering concentration on a NovaSeq6000 instrument using S2 v1 chemistry (Illumina; cat. no. 20028316) in XP mode for the first 3,000 samples and NoveSeq S4 v1.5 chemistry for the last 384 samples, and sequenced paired-end 2*50 cycles before demultiplexing using bcl2fastq2 v2.20. Quality control of the sequencing was evaluated through FastQC (v0.11.9). To quantify the expression of genes in RNA-Seq data, the sequencing reads were first aligned to the human reference genome GRCh38.p13 provided by Ensembl using STAR (v2.7.1). The count matrix was generated with STAR-quantMode GeneCounts using the human gene annotation version GRCh38.101. Genes with an overall mean expression greater than 15 reads and expressed in at least 5% of the participants were used for the following analyses. Finally, in order to normalise for library size and to log-transform the raw data, we applied the varianceStabilizingTransformation function from the DESeq2 R package (v1.30.1).

Among the 1,543 *cis*-genes for the 14 top CpGs, 620 genes were present in our gene expression data. Out of 389 DMR-mapped genes, 183 were present in our data.

**MethReg Integrative Analysis**

To create CpG-TF-target gene triplets, we first linked a given CpG to transcription factors (TFs) with binding sites within ±250 bp of the CpG, using information from the ReMap2020 database, which contains regulatory regions for over a thousand transcriptional regulators obtained using genome-wide DNA-binding experiments such as ChIP-seq. Next, we linked a CpG to a gene if it was located within the gene’s promoter region (< ±2 kb from the transcription start site). For CpGs in distal regions, we linked them either to genes within 500 kb or to the five nearest upstream and five nearest downstream genes from the CpG location. The CpG-TF pairs were then combined with CpG-target gene pairs to create triplets of CpG-TF-target genes. We used the same matched methylation-RNA samples from the Rhineland study. TF activities were estimated using the GSVA R package. The MethReg analyses were performed using the MethReg R package (v.1.14.0)^1^.

**Genomics and Bidirectional Two-sample Mendelian Randomisation (MR)**

Blood samples were genotyped using the Illumina Omni-2.5 exome array, which contains 2,612,357 single-nucleotide polymorphisms (SNPs). Subsequently, genotype data were processed using GenomeStudio (v.2.0.5), and quality controlled with PLINK (v.1.9). SNPs were excluded if not meeting the Hardy-Weinberg disequilibrium criterion (p-value < 1E-5), having a minor allele frequency < 0.01, or showing a poor genotyping rate (< 99%). Additionally, participants were excluded because of a poor call rate of less than 95% (n = 51), abnormal heterozygosity (n = 100), cryptic relatedness (n = 472), or a sex mismatch (n = 43). To account for variation in population structure, which may otherwise cause systematic differences in allele frequencies^2^, we used EIGENSTRAT (v.16000). EIGENSTRAT uses principal components to detect and correct for population structure, which resulted in the exclusion of an additional 236 participants from non-Caucasian descent. Finally, we imputed missing SNPs with IMPUTE (v.2) ^3^ based on the 1000 Genomes (phase 3) reference panel^4^. We ensured a high imputation quality by only including SNPs with an info score metric > 0.3, which indicates reliable imputation^5^.

To determine the relationship between genetic variation and methylation levels, known as methylation quantitative trait loci (meQTLs), we performed GWASs of the identified CpGs in 6,723 participants of the Rhineland Study in whom both genetic and methylation data were available. The GWAS of each identified CpG was adjusted for age, sex, methylation data batch effects, smoking status and the first ten genetic PCs to account for population structure. The genome-wide significance level was set at p-value < 5e-8.

Next, we performed bidirectional two-sample Mendelian Randomisation (MR) analyses to explore potentially causal relationships between the identified CpGs and LHCV/RHCV. The forward MR analyses were performed using the genetic proxies for the identified CpGs as the exposure and those for LHCV/RHCV as the outcome. In the reverse MR analyses, genetic proxies for LHCV/RHCV were used as the exposure, and those for the identified CpGs as the outcome. For the CpGs, our GWAS summary statistics were used. For LHCV and RHCV, the UK Biobank GWAS summary statistics (n=39,691 samples) were used^6^. To meet MR assumptions, only SNPs that were strongly associated (p-value  < 5 × 10^−8^) with each exposure were selected. Second, we performed linkage-disequilibrium (LD) pruning to select the independent SNPs. The clump function in the R package *TwoSampleMR* (v.0.5.6) *^7^* was used for LD pruning with the following parameters: *r*^2^ = 0.001, a window size of 1,000 kb, and the European population of the 1000 Genomes Project as the reference panel. The data on exposure and outcome were harmonised with regard to effect directions, and palindromic SNPs with minor allele frequency close to 0.5 were removed.

**Dietary Assessment and Diet Quality Scores**

Habitual dietary intake was assessed by a self-administered semi-quantitative food frequency questionnaire (FFQ). The questionnaire was originally developed for the European Prospective Investigation into Cancer and Nutrition (EPIC) study in Potsdam and adapted for the Rhineland Study. Participants were questioned about their habitual intake of 132 food and beverage items in the last 12 months, including standard portion sizes^8^. Depending on the food item, there were between four and eleven frequency options available, ranging from “never” to “11 times per day or more”. Additionally, participants were also asked about fat content of consumed dairy and meat products, as well as the types of fat used for food preparation.

The FFQs were eligible for inclusion in analyses provided that information was available for at least 80% of core food items. Fats used for food preparation or additives to hot beverages were not considered as core food items. Missing data on the FFQ were found for 118 core food items, with a maximum of 31 missing values in one FFQ item. Most food items had only 1 or 2 missing values. To retain all observations with ≥80% of questionnaire completion, missing values on the FFQ were imputed using MissForest. Using SAS 9.4, we computed the sum intake of each food/beverage and estimated macro- and micronutrients, water, and energy intake based on data from the German Food Code and Nutrient Database^9^ (v.3.02). The dietary data were categorised into 17 main food groups and 71 subgroups in accordance with the EPIC SOFT classification scheme.

Diet quality scores including Mediterranean-style diet score (MDS)^10^, Dietary Approaches to Stop Hypertension (DASH)^11^, Mediterranean–DASH Intervention for Neurodegenerative Delay (MIND) diet^12^, the Alternate Healthy Eating Index (AHEI)^13^, the Nordic diet score^14^, EAT-Lancet^15^, plant-based diets as assessed by Plant-based Diet Indexes (i.e. overall PDI, healthful PDI, and unhealthful PDI)^16^ and Dietary Inflammatory Index (DII)^17^ were caculated following previous published methods.

**Association of Identified Baseline Methylation Signatures with Longitudinal Change in Brain Imaging Measures**

For longitudinal analysis, we used follow-up imaging data. Complete follow-up data were available in 2,892 participants. To investigate whether the identified baseline methylation signatures were associated with longitudinal changes in imaging measures, linear mixed-effect models were applied as follows:

$$Y_{i}=\beta_{0}+\beta_{1}\cdot age+\beta_{2}\cdot CpG/DMR+\beta_{3}\cdot age\times CpG/DMR+ b_{0,i}+b_{1,i}\cdot age+\varepsilon_{\iota}$$

Here, *Y_i_* denotes the imaging measure at any time point for participant *i, β_0_* denotes the fixed mean intercept, *β_1_* denotes the fixed effect of age (i.e., the average change rate of the outcome per year across all individuals), *β_2_* is the fixed effect of baseline methylation signatures, *β_3_* is the fixed effect of the interaction between baseline methylation signatures and age, and *ε_i_*  represents the residual error. To account for multiple measurements of the same participant across time, we included a random intercept (*b_0,i_*) and slope (*b_1,i_*) for each participant. The effect of baseline methylation signatures on the average change rate of imaging measures was thus given by the interaction term *β_3_*. The average change rate associated with one standard deviation (SD) increase of baseline methylation signatures was calculated as *β_1_* + *β_3_*. All available brain imaging examinations from any time point were included in the model and contributed towards the coefficients. As with the cross-sectional analysis, we adjusted the models for age, sex, batches, and, in the case of hippocampal volume, for eTIV.

To evaluate the variance explained by methylation markers on the random slope, we compared the variance in the random slope of age in a base model, only adjusted for age, sex, and eTIV, to that in the full model, which included all identified CpG or DMR and their interactions with age. All linear mixed-effect models were analysed with the nlme R package (v3.1-168). Model variances were extracted using the VarCorr function from the same package. The percentage of variance explained was calculated as:

*Explained variance (%) = [1- random slope variance (full model)/random slope variance (base model)]*100*

This approach allowed us to quantify the contribution of baseline methylation signatures to explaining inter-individual differences in the rate of imaging measure changes over time.

**References**

1. Silva TC, Young JI, Martin ER, Chen XS, Wang L. MethReg: estimating the regulatory potential of DNA methylation in gene transcription. *Nucleic Acids Res* 2022; **50**(9): e51.

2. Price AL, Patterson NJ, Plenge RM, Weinblatt ME, Shadick NA, Reich D. Principal components analysis corrects for stratification in genome-wide association studies. *Nat Genet* 2006; **38**(8): 904-9.

3. Howie BN, Donnelly P, Marchini J. A flexible and accurate genotype imputation method for the next generation of genome-wide association studies. *PLoS Genet* 2009; **5**(6): e1000529.

4. Genomes Project C, Auton A, Brooks LD, et al. A global reference for human genetic variation. *Nature* 2015; **526**(7571): 68-74.

5. Verma SS, de Andrade M, Tromp G, et al. Imputation and quality control steps for combining multiple genome-wide datasets. *Front Genet* 2014; **5**: 370.

6. Smith SM, Douaud G, Chen W, et al. An expanded set of genome-wide association studies of brain imaging phenotypes in UK Biobank. *Nat Neurosci* 2021; **24**(5): 737-45.

7. Hemani G, Zheng J, Elsworth B, et al. The MR-Base platform supports systematic causal inference across the human phenome. *Elife* 2018; **7**.

8. Nothlings U, Hoffmann K, Bergmann MM, Boeing H. Fitting portion sizes in a self-administered food frequency questionnaire. *J Nutr* 2007; **137**(12): 2781-6.

9. Dehne LI, Klemm C, Henseler G, Hermann-Kunz E. The German Food Code and Nutrient Data Base (BLS II.2). *Eur J Epidemiol* 1999; **15**(4): 355-9.

10. Trichopoulou A, Costacou T, Bamia C, Trichopoulos D. Adherence to a Mediterranean diet and survival in a Greek population. *N Engl J Med* 2003; **348**(26): 2599-608.

11. Fung TT, Chiuve SE, McCullough ML, Rexrode KM, Logroscino G, Hu FB. Adherence to a DASH-style diet and risk of coronary heart disease and stroke in women. *Arch Intern Med* 2008; **168**(7): 713-20.

12. Morris MC, Tangney CC, Wang Y, et al. MIND diet slows cognitive decline with aging. *Alzheimers Dement* 2015; **11**(9): 1015-22.

13. Chiuve SE, Fung TT, Rimm EB, et al. Alternative dietary indices both strongly predict risk of chronic disease. *J Nutr* 2012; **142**(6): 1009-18.

14. Galbete C, Kroger J, Jannasch F, et al. Nordic diet, Mediterranean diet, and the risk of chronic diseases: the EPIC-Potsdam study. *BMC Med* 2018; **16**(1): 99.

15. Willett W, Rockstrom J, Loken B, et al. Food in the Anthropocene: the EAT-Lancet Commission on healthy diets from sustainable food systems. *Lancet* 2019; **393**(10170): 447-92.

16. Satija A, Bhupathiraju SN, Spiegelman D, et al. Healthful and Unhealthful Plant-Based Diets and the Risk of Coronary Heart Disease in U.S. Adults. *J Am Coll Cardiol* 2017; **70**(4): 411-22.

17. Shivappa N, Steck SE, Hurley TG, Hussey JR, Hebert JR. Designing and developing a literature-derived, population-based dietary inflammatory index. *Public Health Nutr* 2014; **17**(8): 1689-96.
