# Supplementary material for: DNA methylation signatures of bilateral hippocampal volume, asymmetry and atrophy: a cross-omics analysis in the general population
Source: eBioMedicine. 2026 May 11;128:106289. doi: 10.1016/j.ebiom.2026.106289 (PMC13191633; doi:10.1016/j.ebiom.2026.106289)
Supplement: Supplementary Figures [file mmc2.docx]

**DNA methylation signatures of bilateral hippocampal volume, asymmetry and atrophy: a cross-omics analysis in the general population**

**D. Liu et al.**

**Supplementary Figures 1-15**

**Figure S1.** Overview of the Rhineland Study datasets used in the integrative multiomics analysis and follow-up analyses

**Figure S2.** Scree plot of the genetic principal components

**Figure S3.** Distribution of hippocampal and global grey matter volume and asymmetry across age and sex in the Rhineland Study

**Figure S4.** Upset plots show the identified CpGs overlap among models

**Figure S5.** Manhattan plots of the epigenome-wide meta-analyses of hippocampal and global grey matter volume and asymmetry adjusted for handedness

**Figure S6.** The overlap genes between genes detected by differentially methylated region analysis among hippocampal and grey matter volume and asymmetry

**Figure S7.** Shared gene ontology pathways identified for hippocampal and grey matter volume and asymmetry

**Figure S8.** KEGG pathways identified for hippocampal and grey matter volume and asymmetry

**Figure S9.** Specific gene ontology pathways identified for hippocampal and grey matter volume and asymmetry

**Figure S10.** Pathway enrichment of hypo- vs. hypermethylated CpGs

**Figure S11.** GWAS traits previously associated with key genes

**Figure S12.** Genomic annotation of identified methylation signatures

**Figure S13.** The association of CpGs/DMR with putative transcription factors and target gene expressions

**Figure S14.** Manhattan plot showing the genome-wide signals of hippocampal-related CpGs

**Figure S15.** Bidirectional two-sample Mendelian Randomisation analyses reveal causal relationships between identified CpG and right hippocampal volume

**Figure S1. Overview of the Rhineland Study datasets used in the integrative multiomics analysis and follow-up analyses**


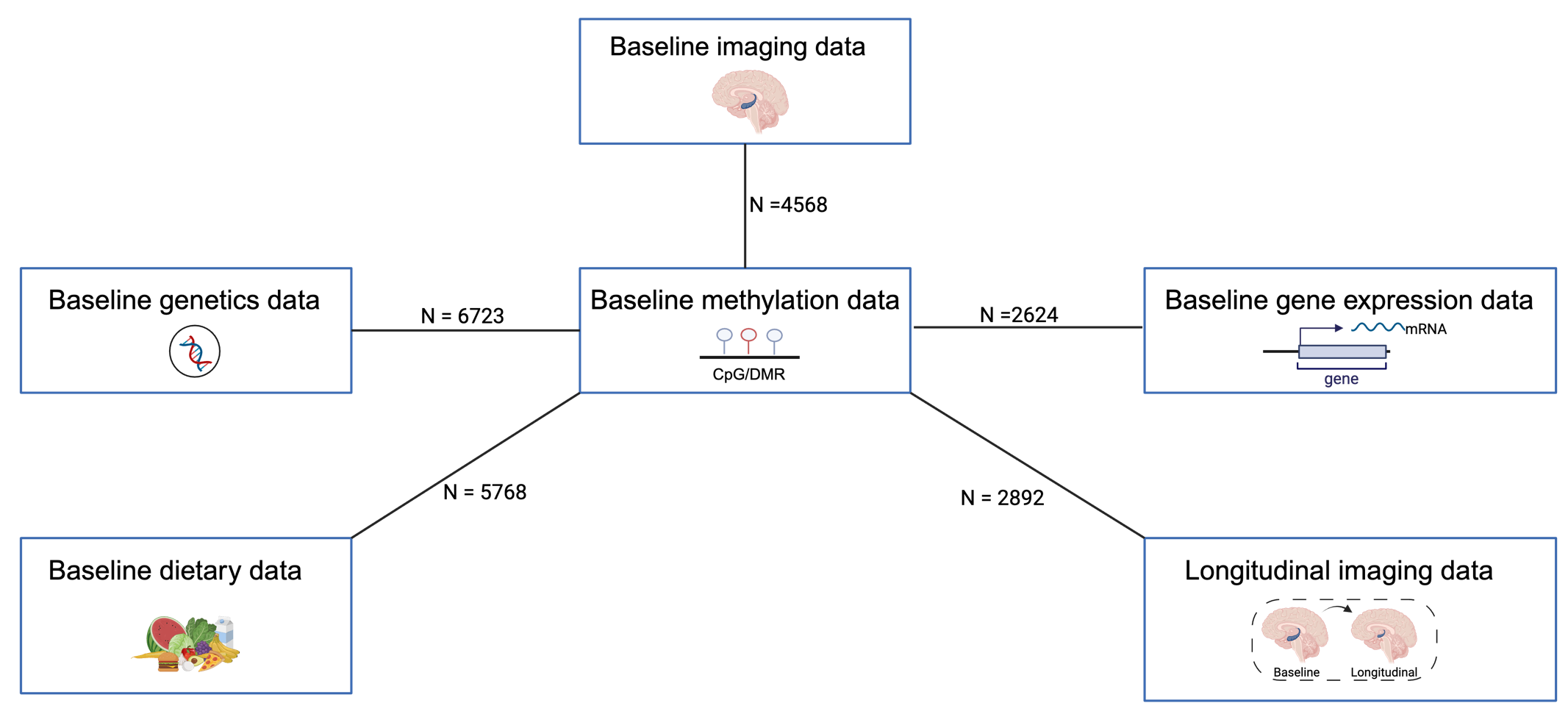


The numbers indicate the sample size available for the assessment of each association.

**Figure S2. Scree plot of the genetic principal components**

**
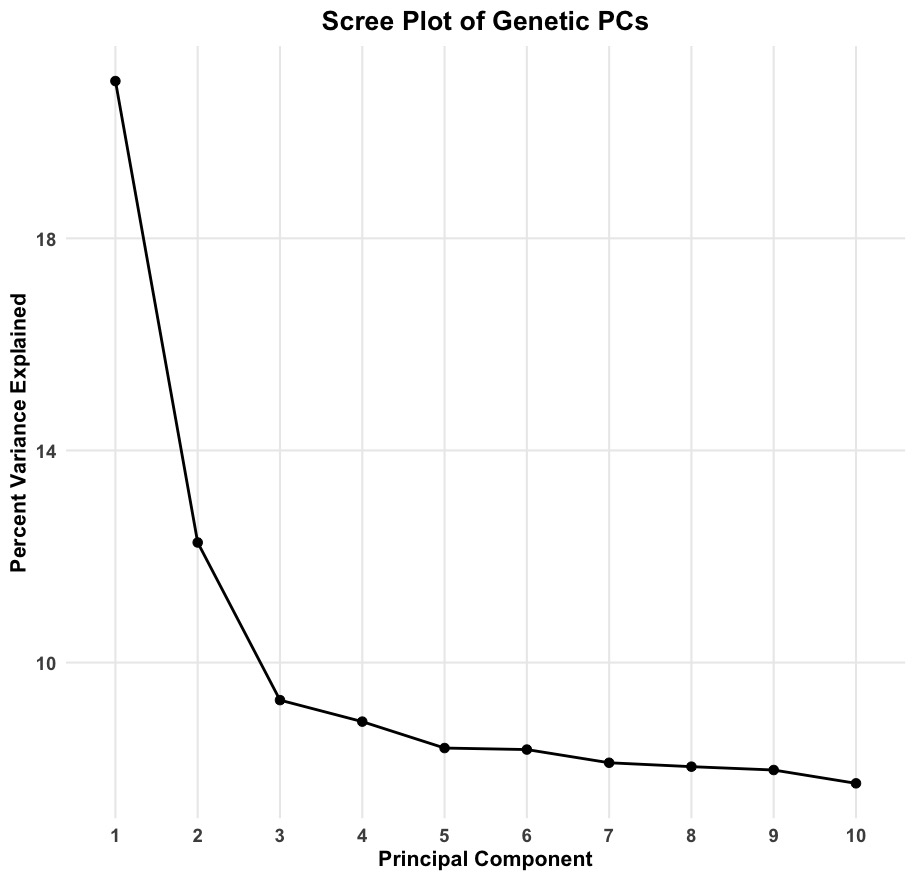
**

**Figure S3. Distribution of hippocampal and global grey matter volume and asymmetry across age and sex in the Rhineland Study**

Scatterplots of associations between brain-imaging-derived endophenotypes and age (sample size n = 4568). Regression lines for volumetric measures were adjusted for age, sex and estimated total intracranial volume. Regression lines for asymmetry traits were adjusted for age and sex.

Abbreviations: f, female; m, male; LHCV and RHCV, left and right hippocampal volumes; LGMV and RGMV, left and right hemisphere grey matter volumes; HCasy, hippocampal asymmetry; GMasy, global grey matter asymmetry; eTIV, estimated total intracranial volume.

**Figure S4. Upset plots show the identified CpGs overlap among models**

**
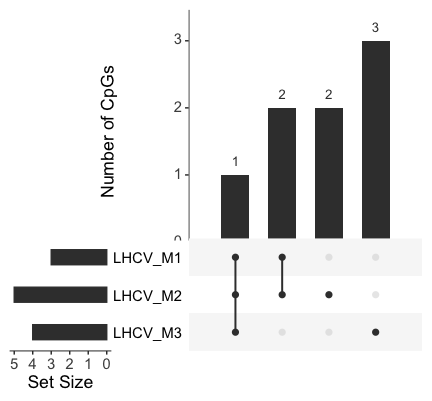
**
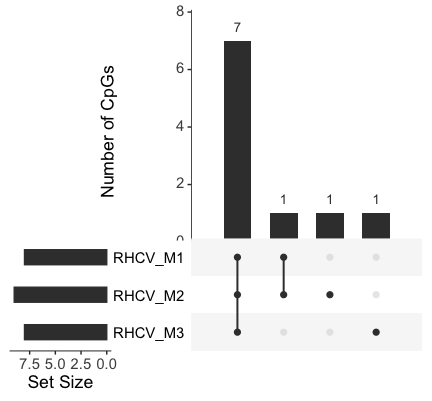


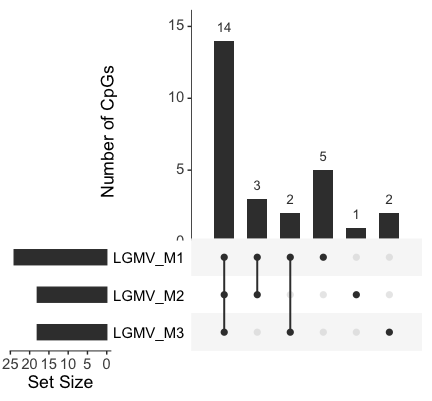
 **
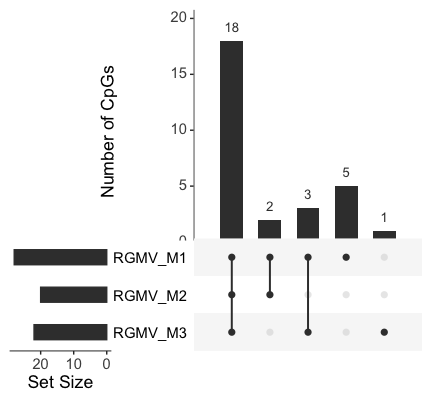
**

Abbreviations: LHCV and RHCV, left and right hippocampal volumes; LGMV and RGMV, left and right hemisphere grey matter volumes

M1, model 1; M2, model 2, M3, model 3.

**Figure S5. Manhattan plots of the epigenome-wide meta-analyses of hippocampal and global grey matter volume and asymmetry adjusted for handedness**


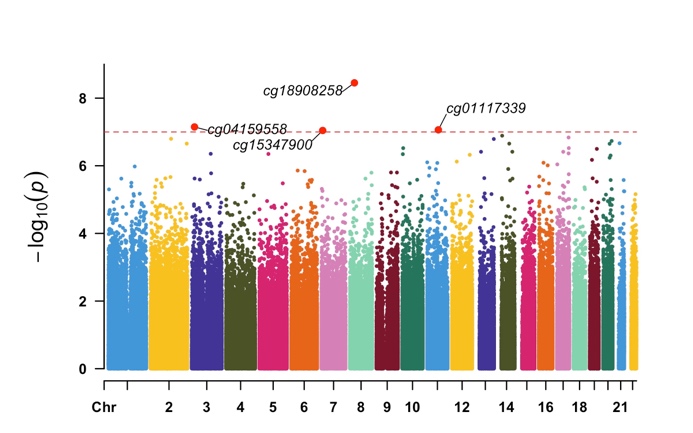

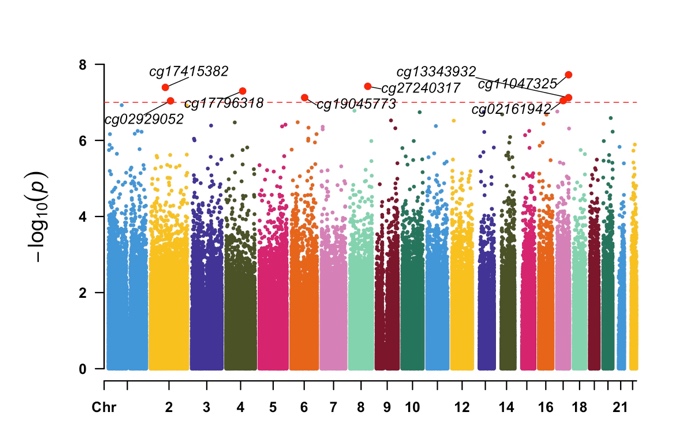

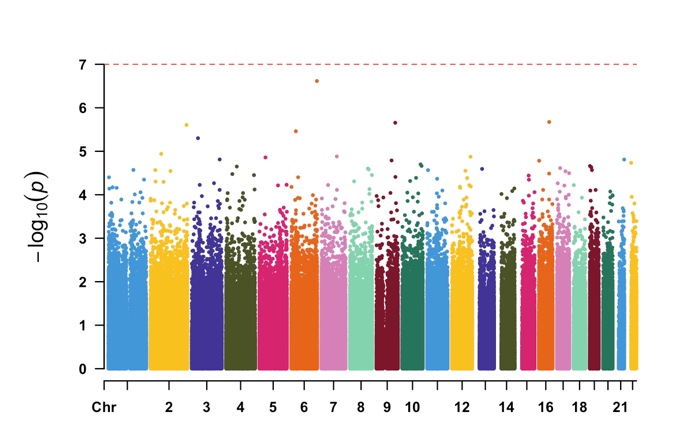


**LHCV RHCV Hippocampal asymmetry**


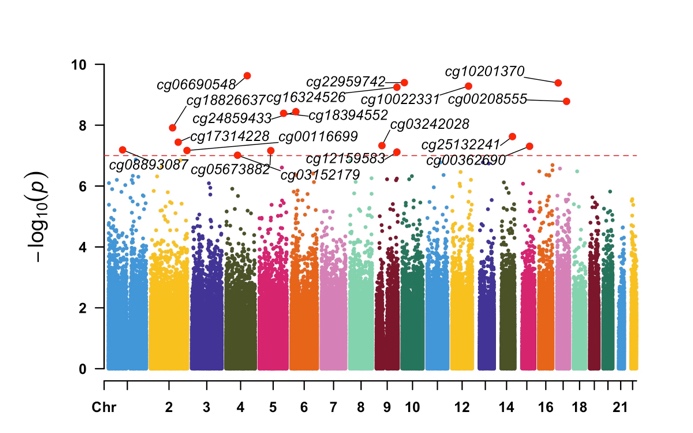

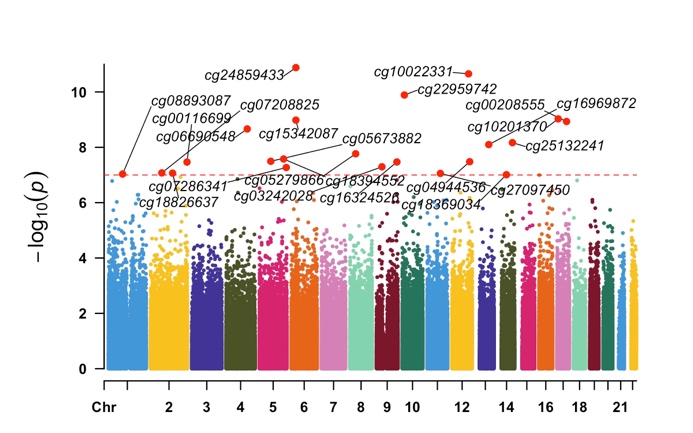

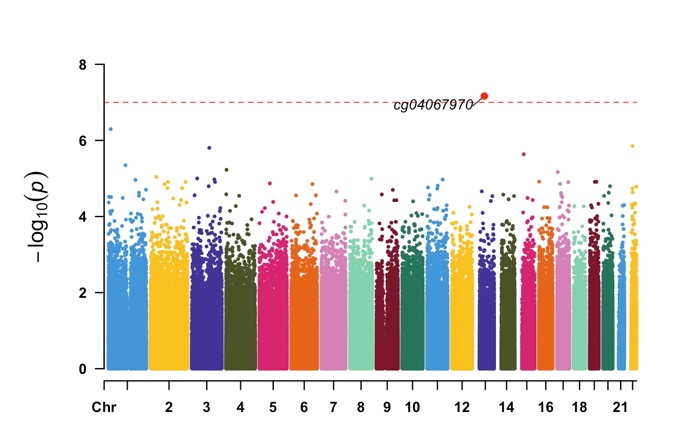


**LGMV RGMV Grey matter asymmetry**

Results were plotted as negative log-transformed p-values (y-axis) across the genome (x-axis) (sample size n = 8,156). The red horizontal line represents the epigenome-wide significance at 1.0 x10^-7^. Linear models were adjusted for age, sex, estimated total intracranial volume, batch effects, blood cell proportion, the first ten genetic principal components (to account for population stratification), smoking status, education and handedness. All the epigenome-wide significant CpGs were annotated.

Abbreviations: LHCV and RHCV, left and right hippocampal volumes; LGMV and RGMV, left and right hemisphere grey matter volumes.

**Figure S6. The overlap genes between genes detected by differentially methylated region analysis among hippocampal and grey matter volume and asymmetry**


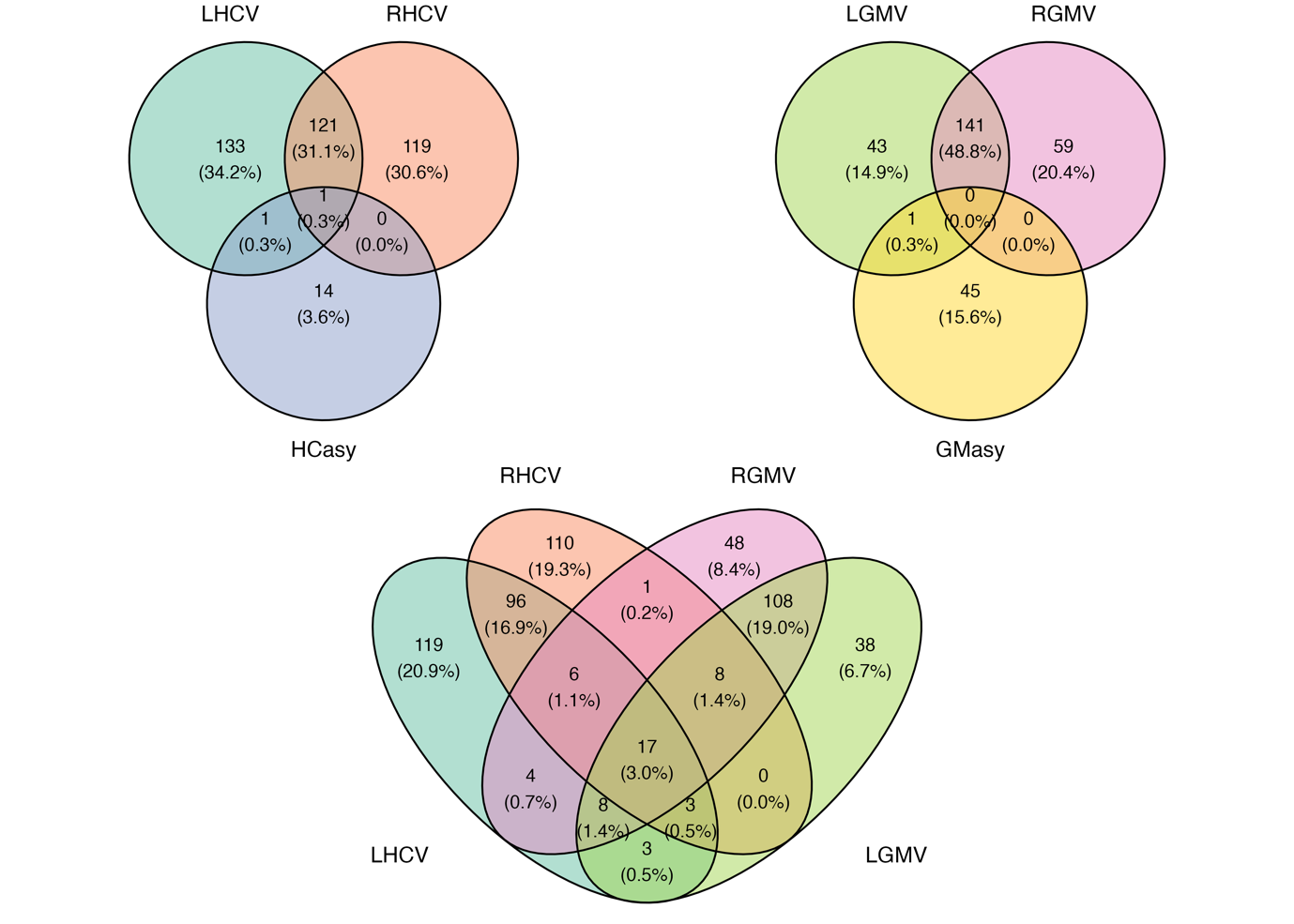


Abbreviations: LHCV and RHCV, left and right hippocampal volumes; LGMV and RGMV, left and right hemisphere grey matter volumes; HCasy, hippocampal asymmetry; GMasy, global grey matter asymmetry.

**Figure S7. Shared gene ontology pathways identified for hippocampal and grey matter volume and asymmetry**

**
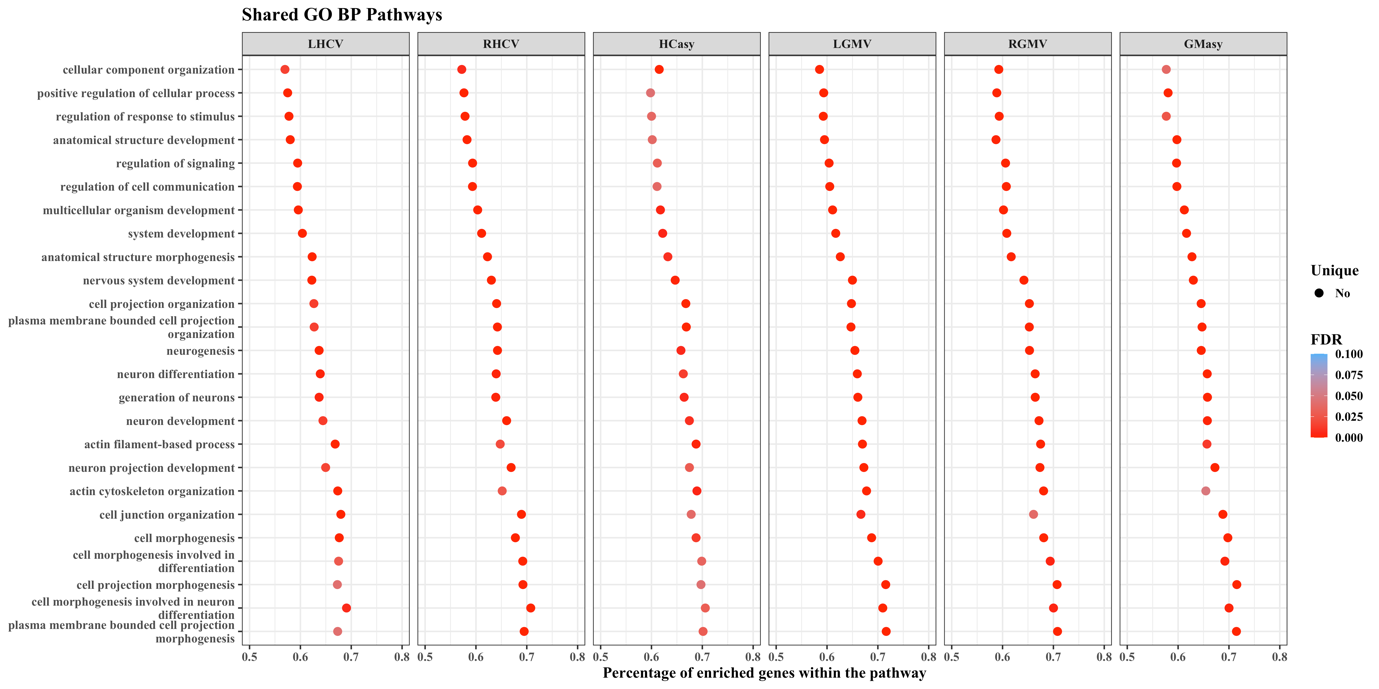
**

**
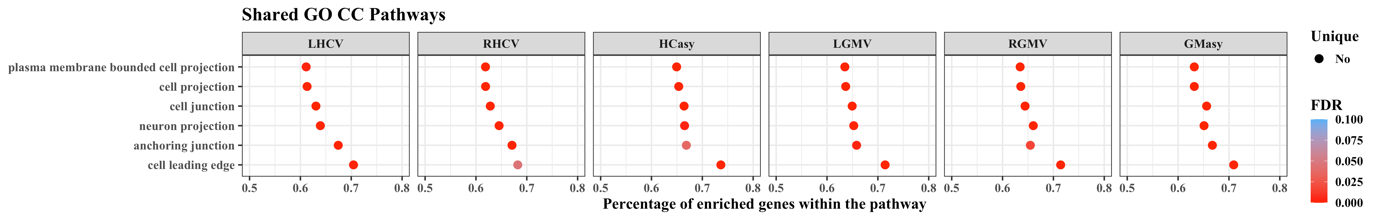
**

**
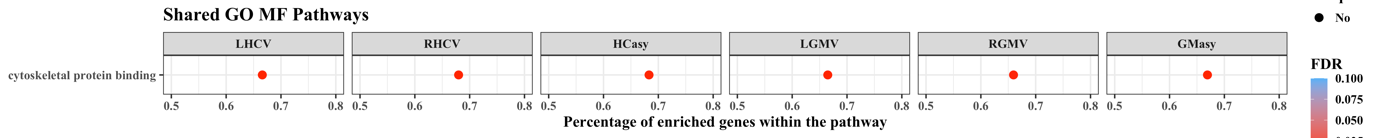
**

Abbreviations: LHCV and RHCV, left and right hippocampal volumes; LGMV and RGMV, left and right hemisphere grey matter volumes; HCasy, hippocampal asymmetry; GMasy, global grey matter asymmetry. GO: gene ontology database

**Figure S8. KEGG pathways identified for hippocampal and grey matter volume and asymmetry**

**
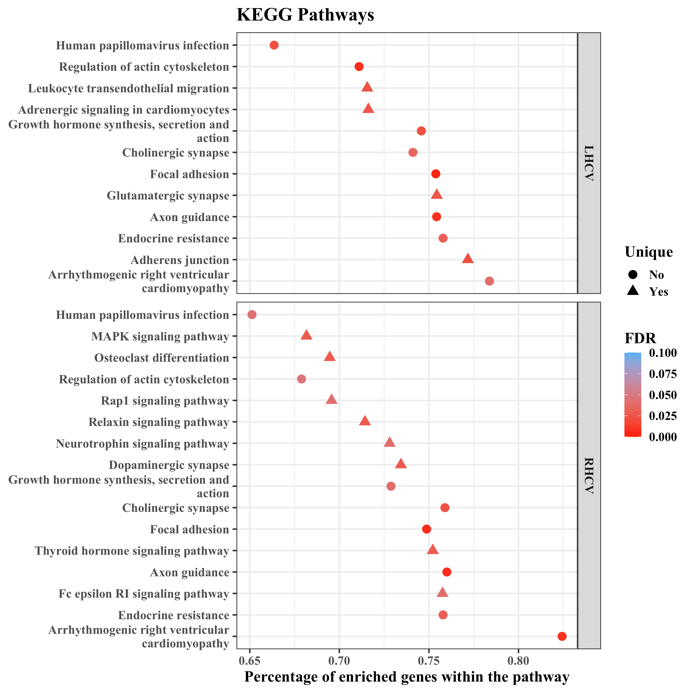

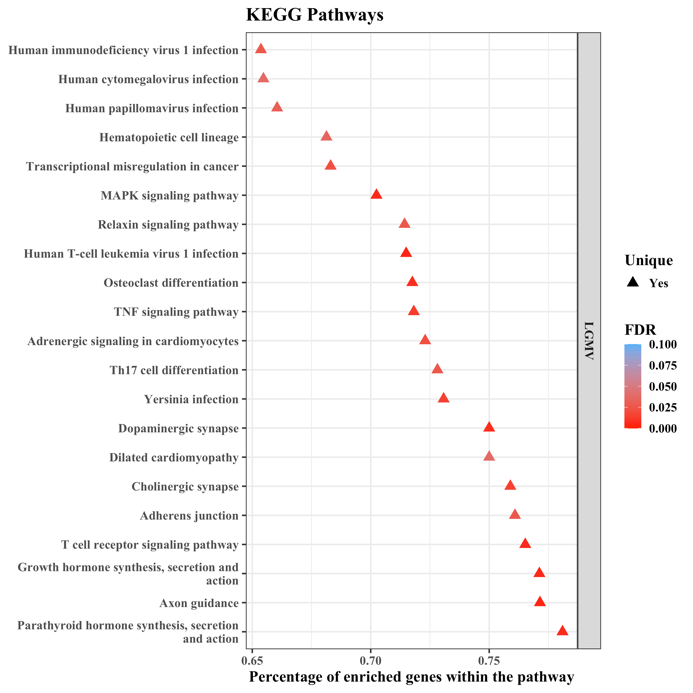

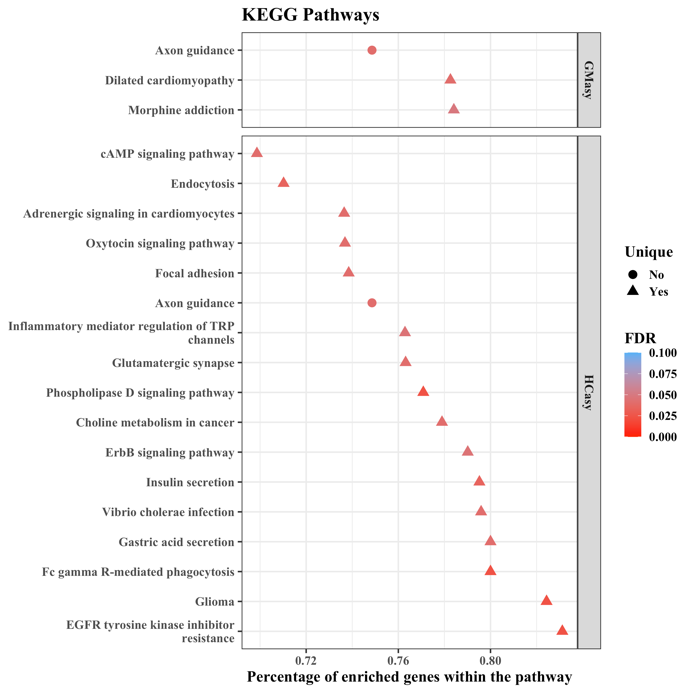
**

Abbreviations: LHCV and RHCV, left and right hippocampal volumes; LGMV and RGMV, left and right hemisphere grey matter volumes; HCasy, hippocampal asymmetry; GMasy, global grey matter asymmetry; FDR, false discovery rate; KEGG, Kyoto Encyclopedia of Genes and Genomes.

**Figure S9. Specific gene ontology pathways identified for hippocampal and grey matter volume and asymmetry**

**
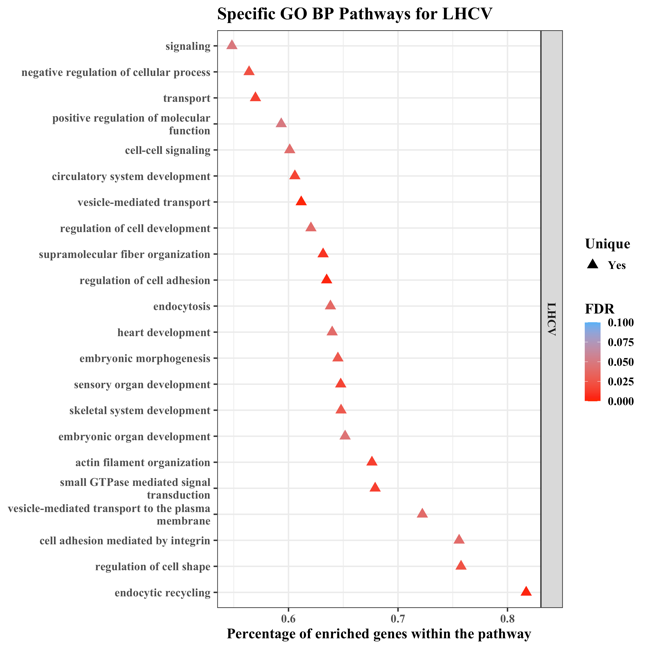

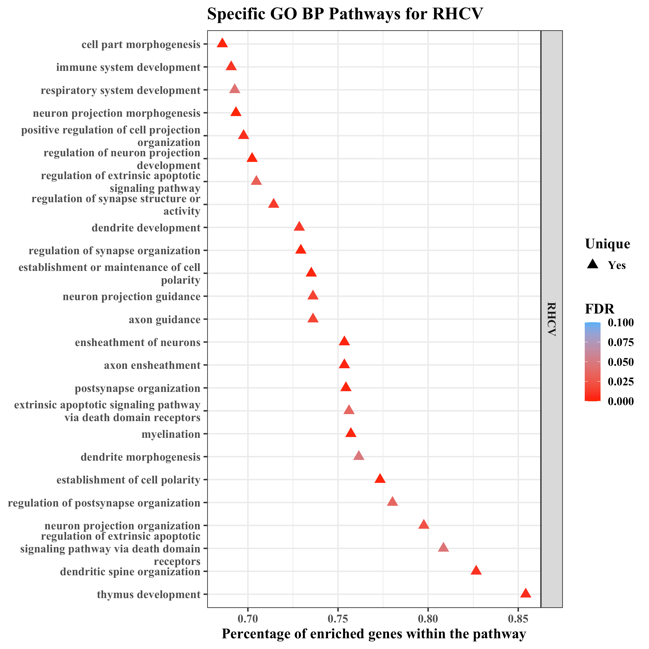
**

**
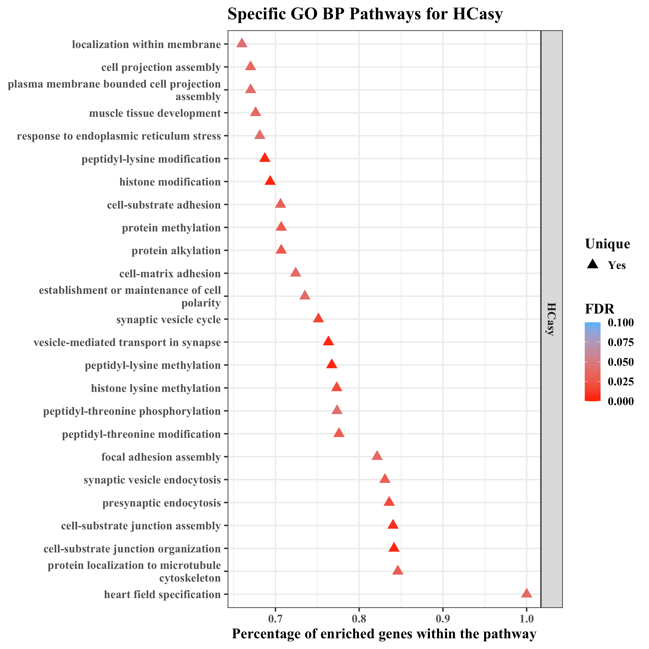

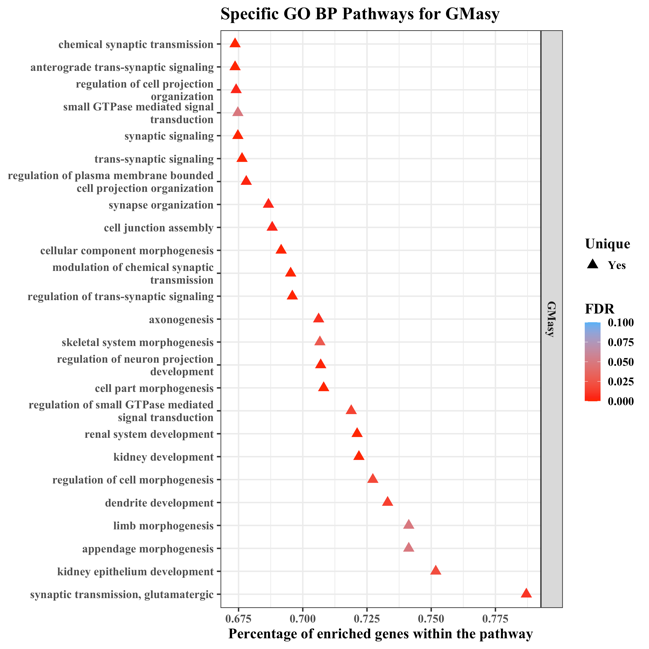
**

**
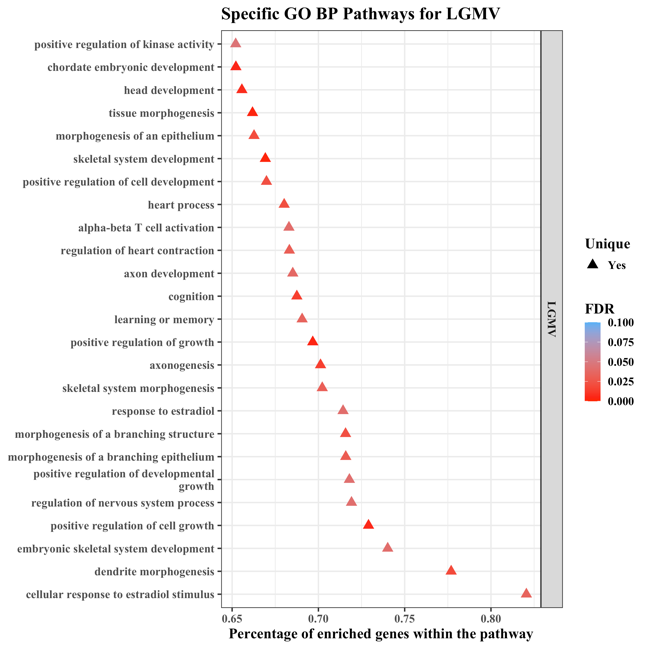

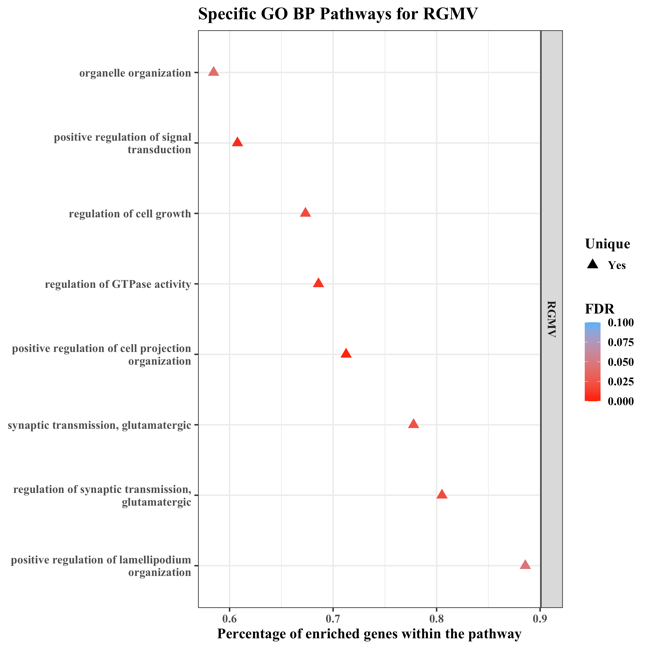
**

Abbreviations: LHCV and RHCV, left and right hippocampal volumes; LGMV and RGMV, left and right hemisphere grey matter volumes; HCasy, hippocampal asymmetry; GMasy, global grey matter asymmetry; GO BP: gene ontology biological process database; FDR, false discovery rate.

**Figure S10: Pathway enrichment of hypo- vs. hypermethylated CpGs**

**
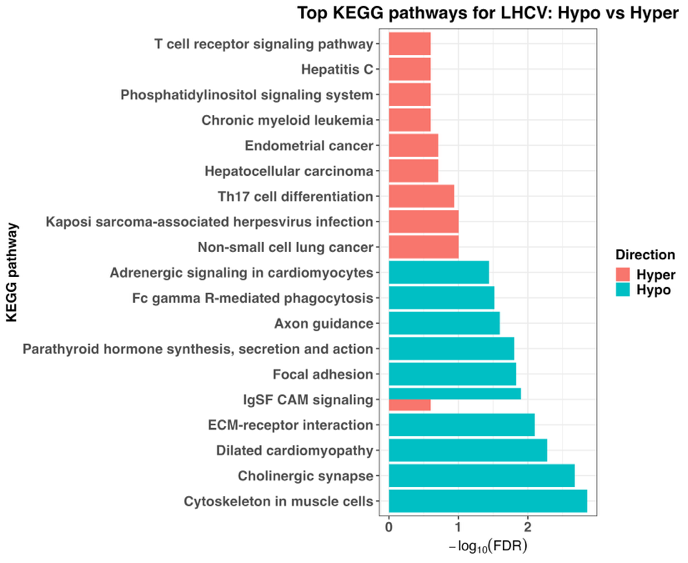

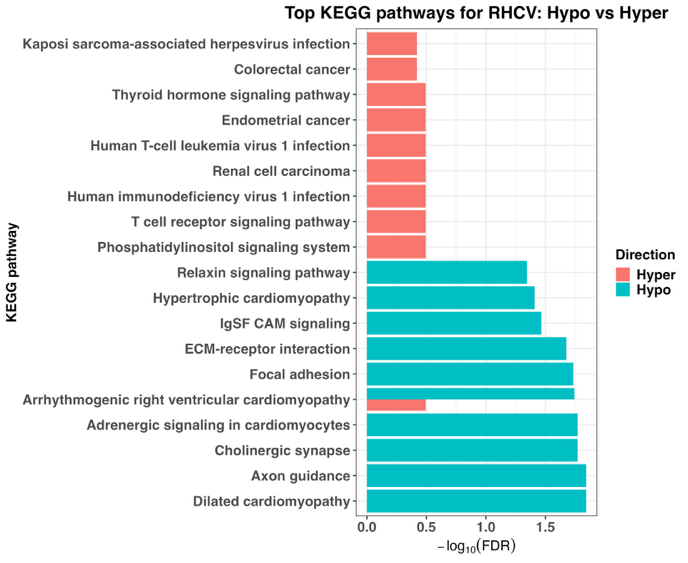
**

**
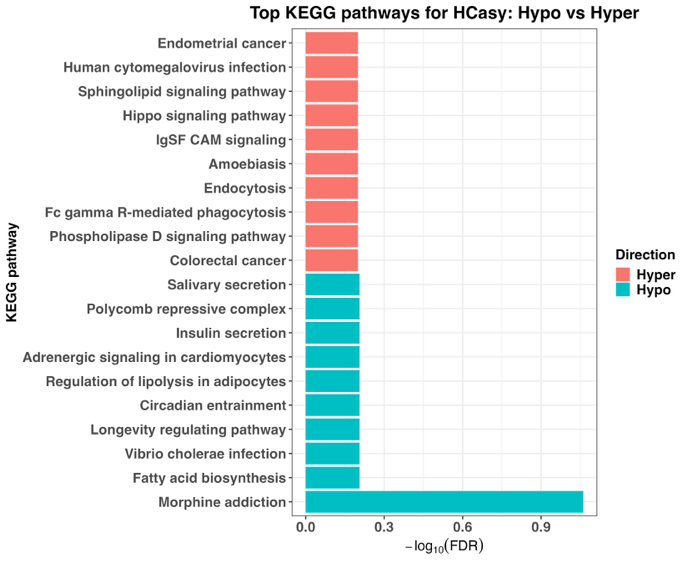
**

Abbreviations: LHCV and RHCV, left and right hippocampal volumes; HCasy, hippocampal asymmetry; FDR, false discovery rate; KEGG, Kyoto Encyclopedia of Genes and Genomes

**Figure S11. GWAS traits previously associated with key genes**

Key genes implicated in LHCV


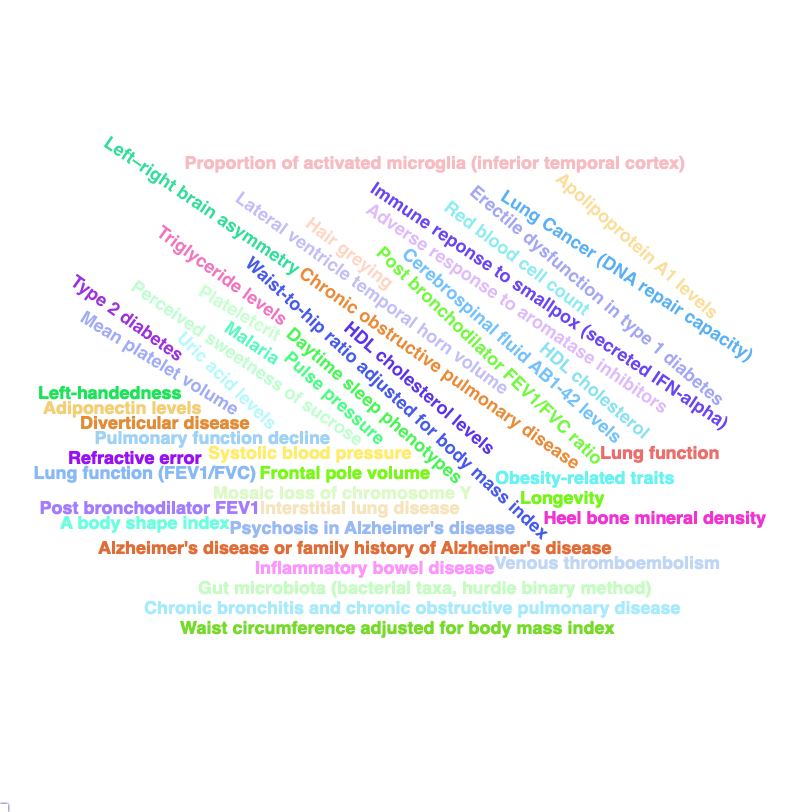


Key genes implicated in RHCV


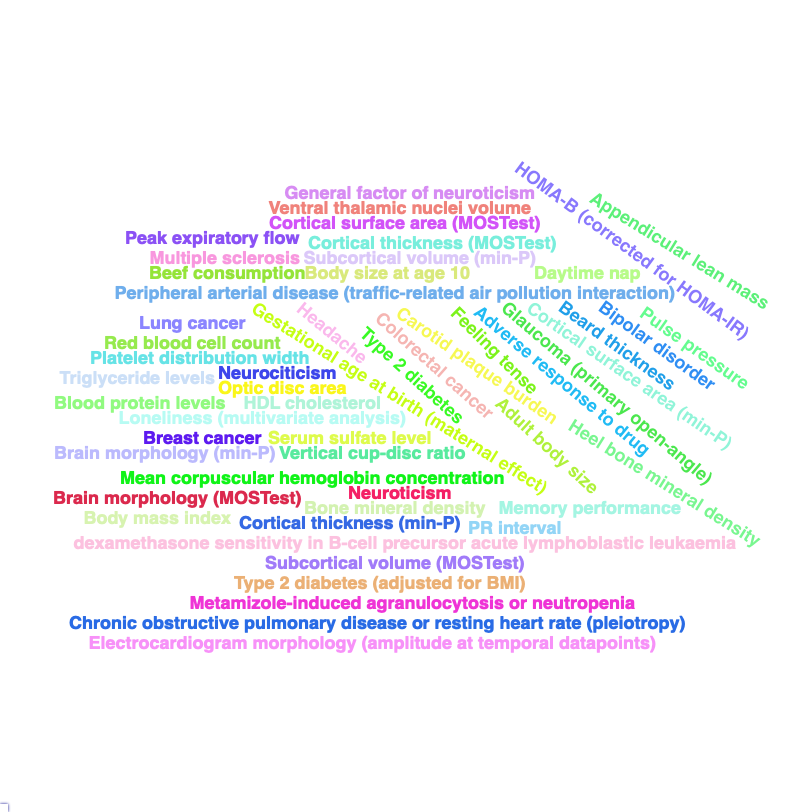


Key genes implicated in hippocampal asymmetry


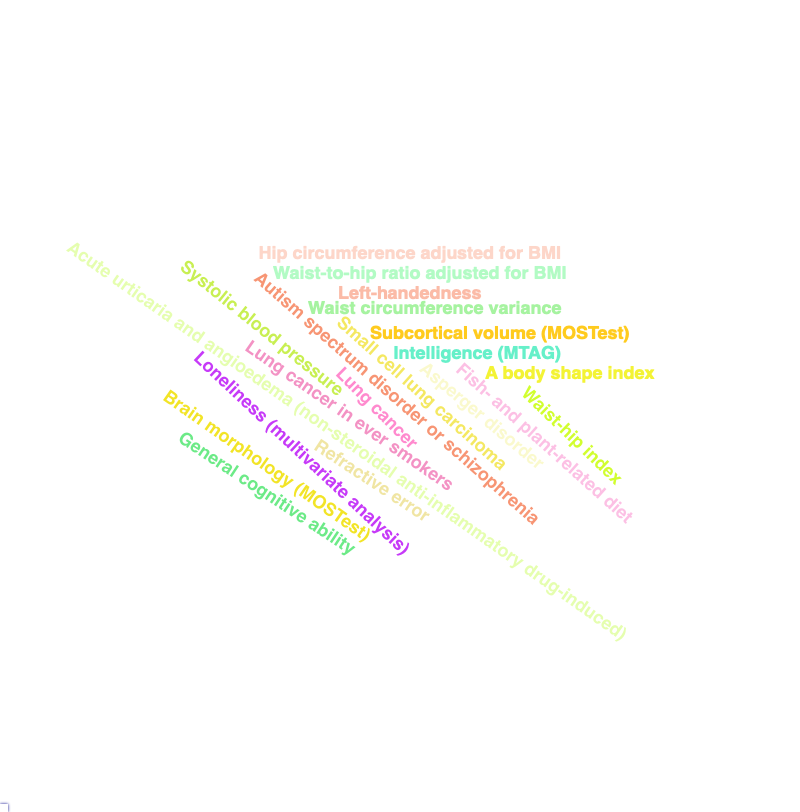


**Figure S12. Genomic annotation of identified methylation signatures**

1. LHCV


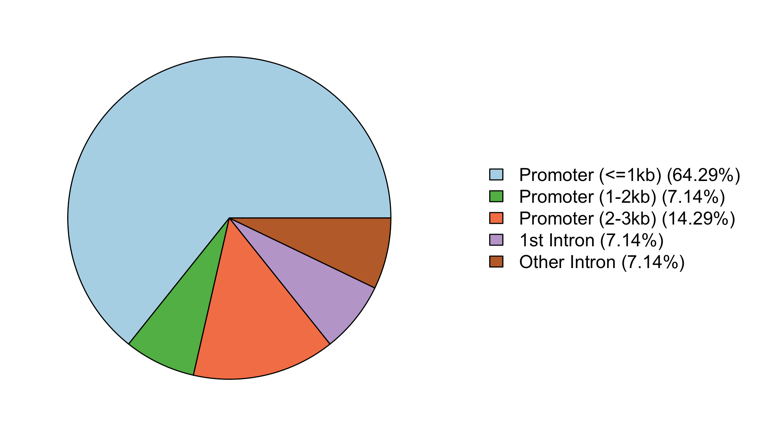

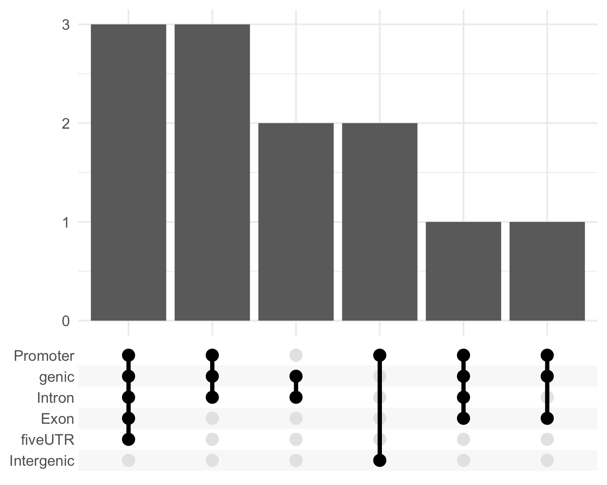


1. RHCV


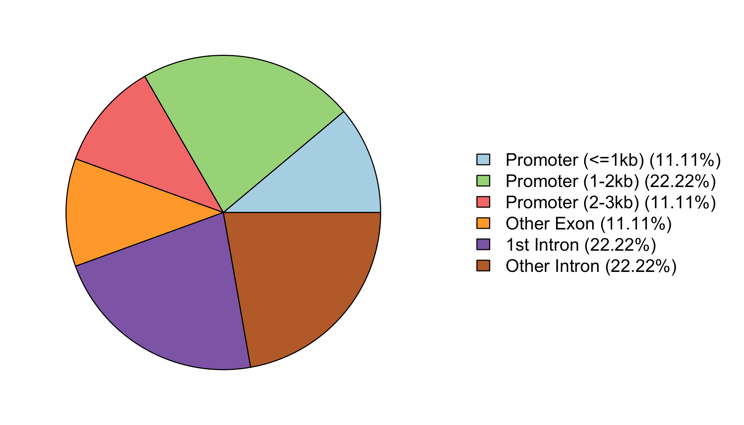

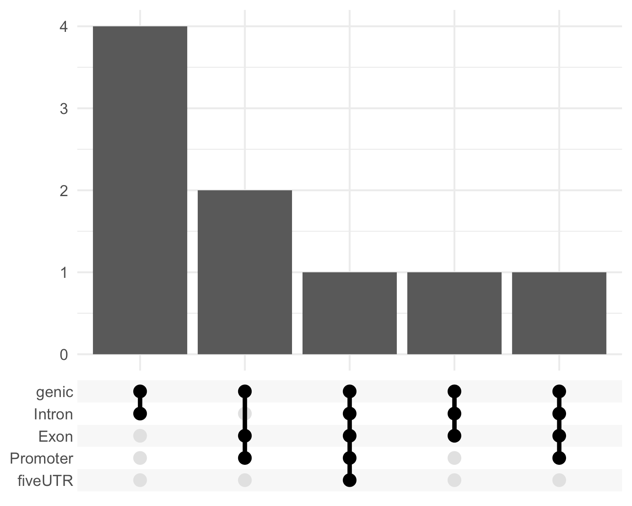


Genomic annotation of CpGs and DMRs loci associated with LHCV (A) and RHCV (B). The pieplots and the upset plots were generated using ChIPseeker v1.26.2 R package.

Abbreviations: LHCV and RHCV, left and right hippocampal volumes.

**Figure S13. The association of CpGs/DMR with putative transcription factors and target gene expressions**

**
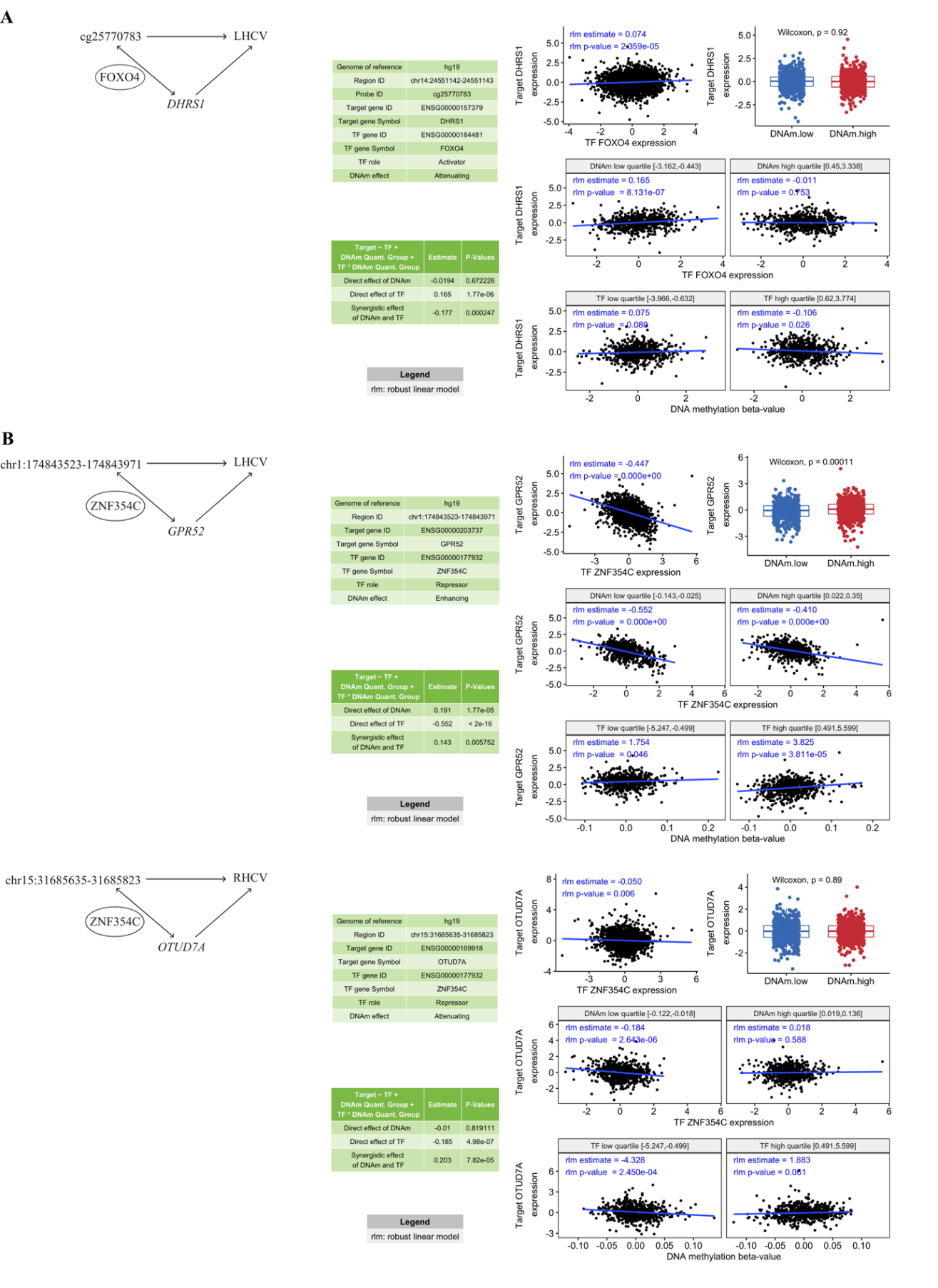
**

**
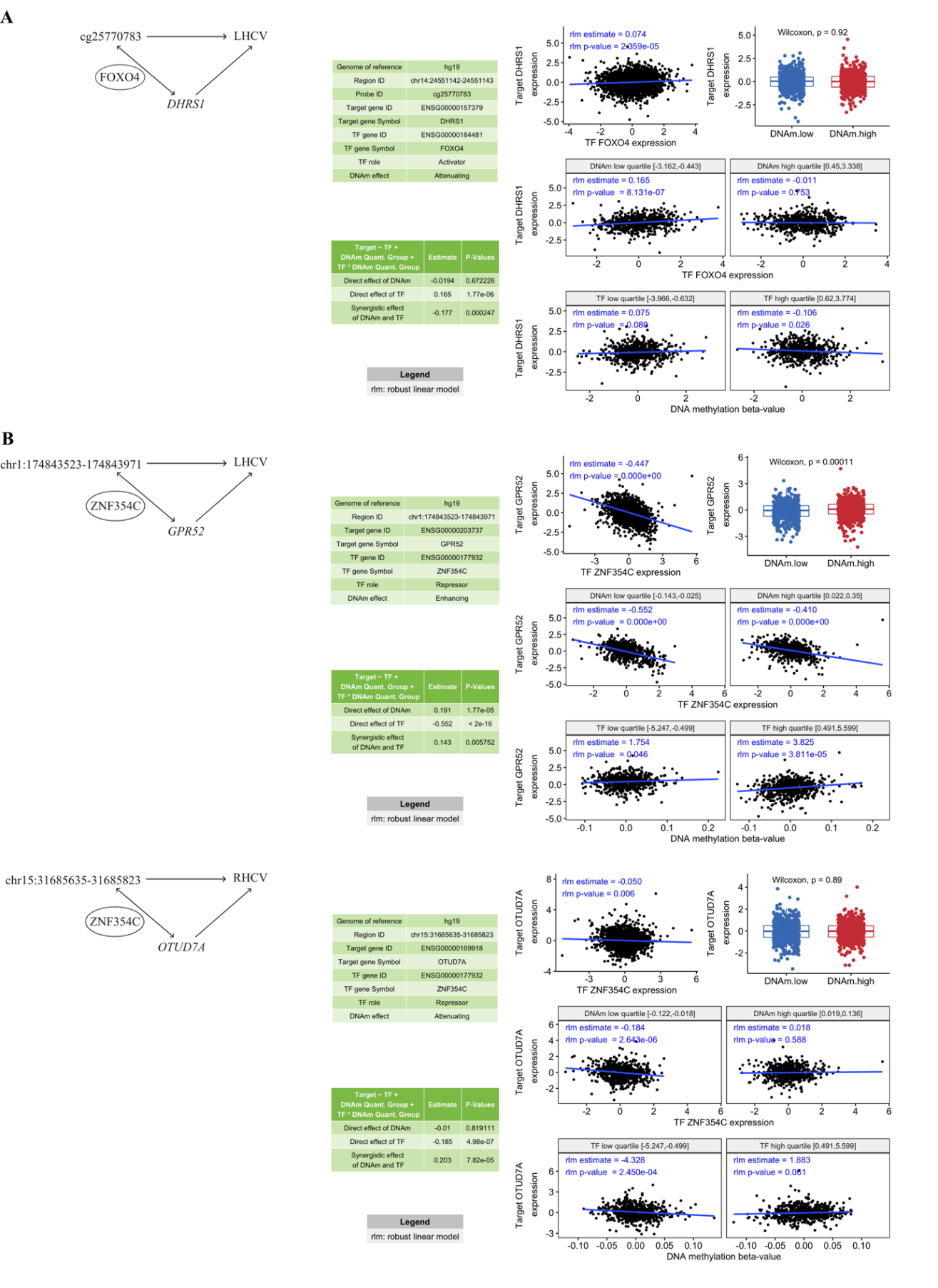
**

Results of MethReg analysis. The plots show the relationship between DNA methylation and target gene (1st row), the relationship between transcription factor and target genes in low and high DNA methylation level groups (2nd row) and the relationship between DNA methylation and target gene in low and high transcription factor level groups (3rd row). (A) Results for cg25770783 – FOXO4 – DHRS1 triplet for which the interaction term between cg25770783 and FOXO4 was significant. cg25770783 attenuated FOXO4 activity. (B) Results for two triplets for which the interaction terms between DMR and transcription factor, as well as the association between the target gene and the related trait, were significant. In the “chr1:174843523-174843971 - ZNF354C - GPR52” triplet, the DMR enhances the transcription factor activity. In the “chr15:31685635-31685823 - ZNF354C - OTUD7A” triplet, the DMR attenuates the transcription factor activity.

**Figure S14. Manhattan plot showing the genome-wide signals of hippocampal-related CpGs**

1. GWAS of LHCV-related CpGs


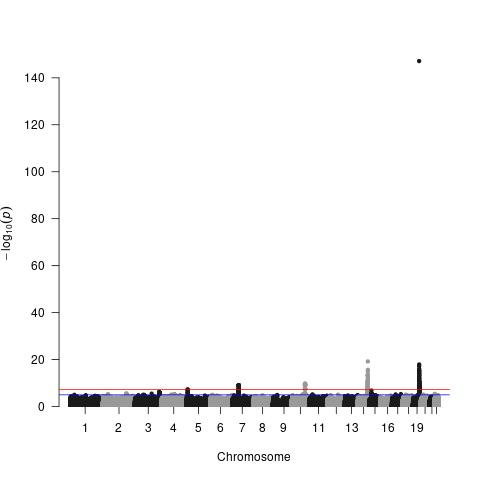

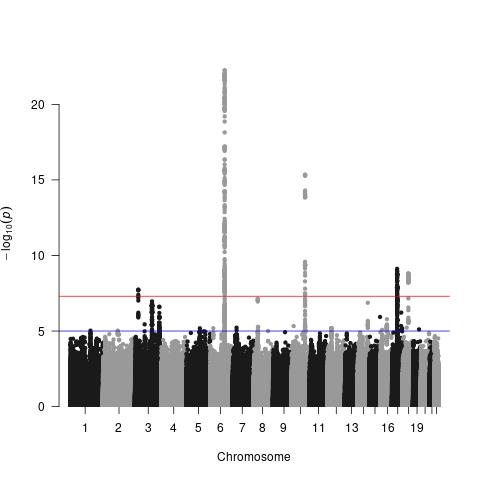

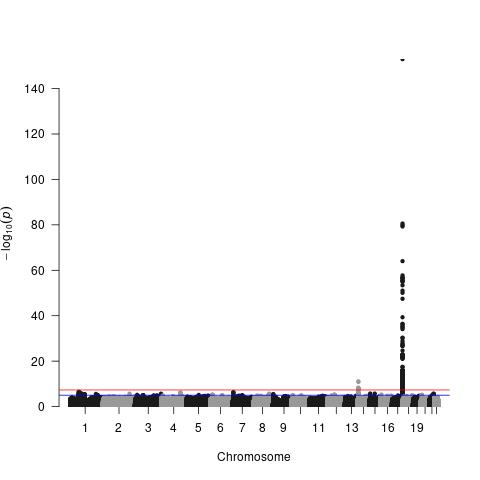


cg03826366 cg13343932 cg25770783

1. GWAS of RHCV-related CpGs

**
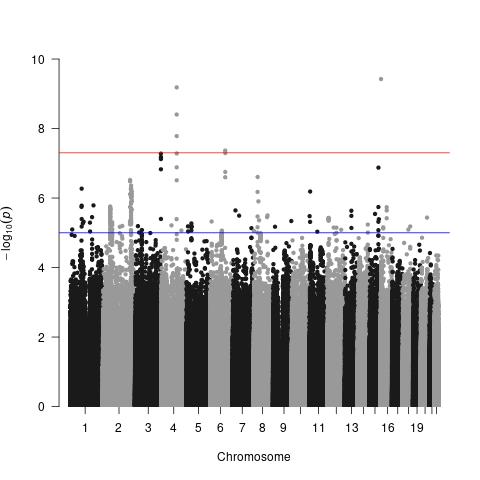

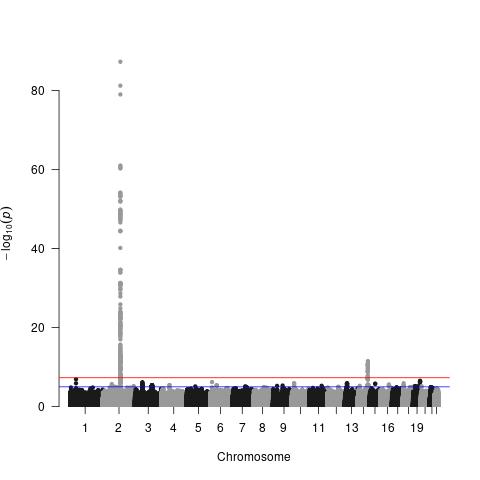

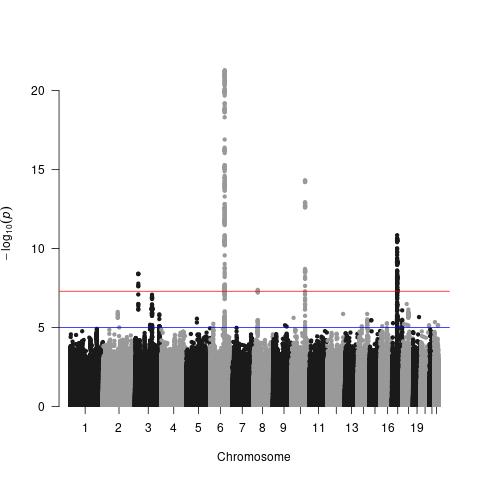
**

cg02348995 cg02929052 cg11047325

**
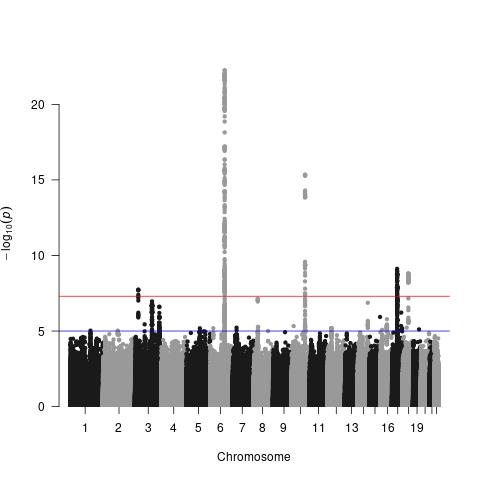

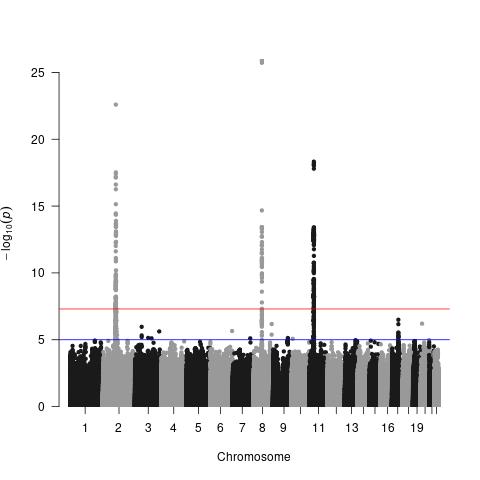

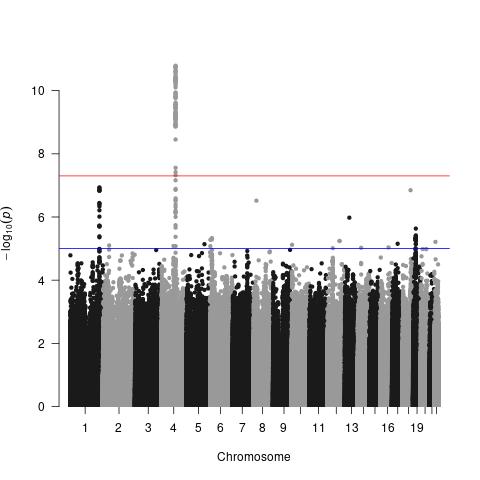
**

cg13343932 cg17415382 cg17796318

**
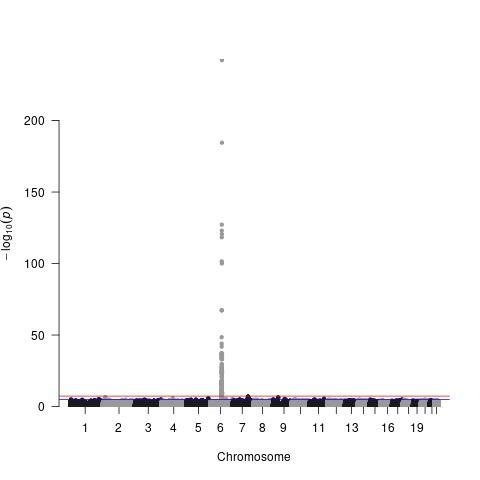

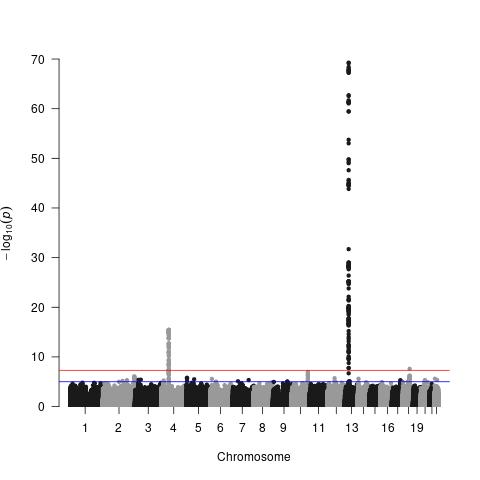

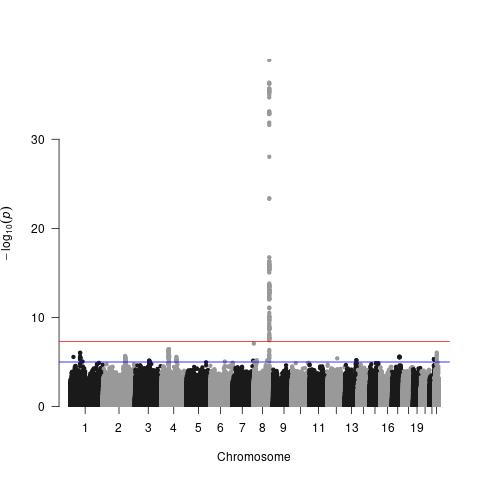
**

cg19045773 cg19122233 cg27240317

**Figure S15. Bidirectional two-sample Mendelian Randomisation analyses reveal causal relationships between identified CpG and right hippocampal volume**


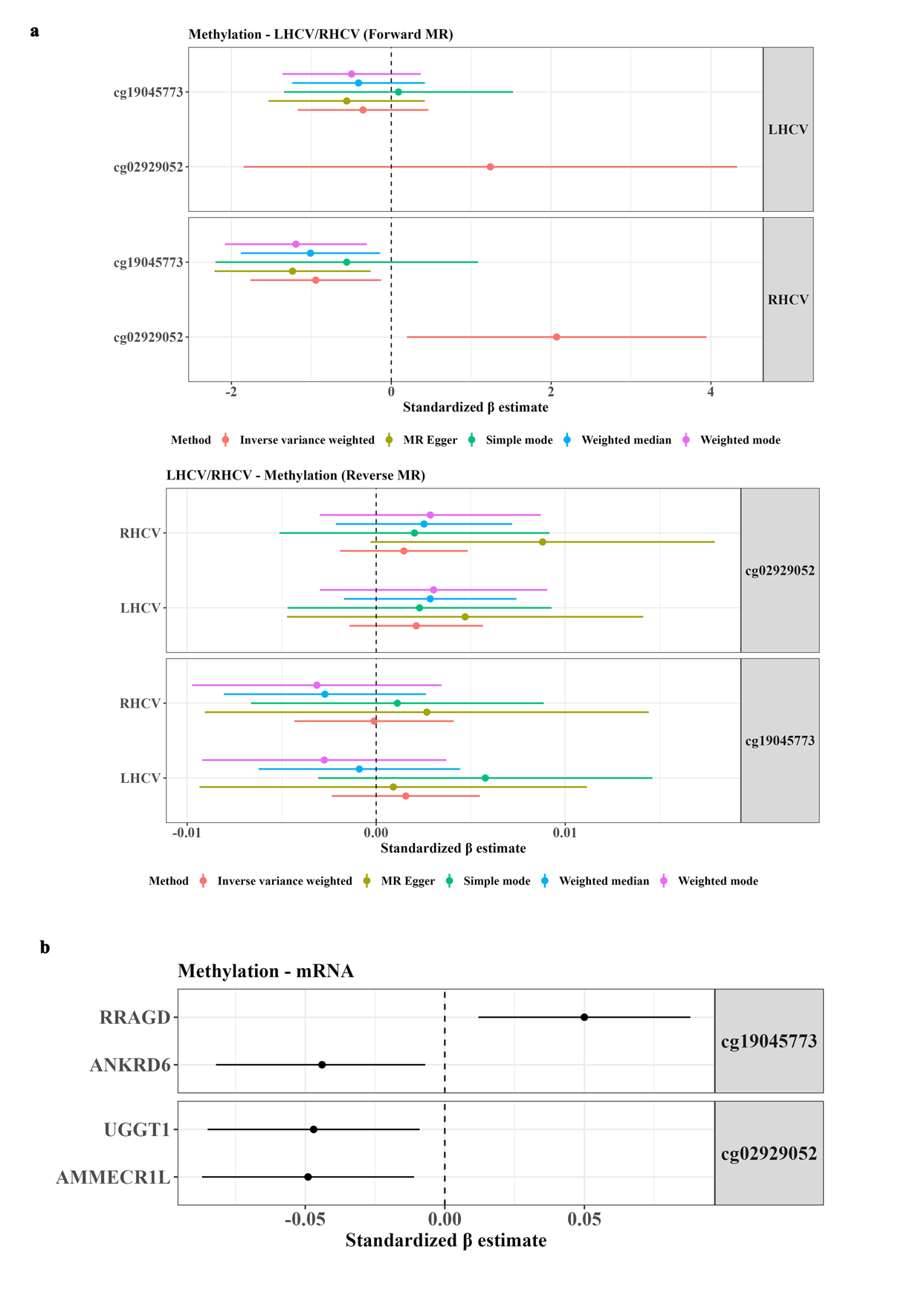


Methylation sites cg19045773 and cg0292052 were identified to be specifically associated with RHCV in the EWAS meta-analysis. **(a)** Forest plots show the causal effect estimates of cg19045773 and cg0292052 on LHCV and RHCV from forward MR analysis, as well as the reverse effects of LHCV and RHCV on methylation.

**(b)** Forest plots show the effect of cg19045773 and cg0292052 on mRNA expression levels. The dot represents the mean effect, and the horizontal line shows the 95% CI. Abbreviations: LHCV and RHCV, left and right hippocampal volumes; MR, Mendelian Randomisation.
